# A cell-permeable nanobody to restore F508del cystic fibrosis transmembrane conductance regulator activity

**DOI:** 10.1038/s41589-026-02199-w

**Published:** 2026-04-17

**Authors:** Luise Franz, Tihomir Rubil, Anita Balázs, Marie Overtus, Kristin Kemnitz-Hassanin, Cedric Govaerts, Marcus A. Mall, Christian P. R. Hackenberger

**Affiliations:** 1 Leibniz-Forschungsinstitut für Molekulare Pharmakologie (FMP), Berlin, Germany; 2 Institute of Chemistry and Biochemistry, Freie Universität Berlin, Berlin, Germany; 3 Department of Pediatric Respiratory Medicine, Immunology and Critical Care Medicine, Charité − Universitätsmedizin Berlin, Berlin, Germany; 4 German Center for Lung Research (DZL), associated partner site Berlin, Berlin, Germany; 5 German Center for Child and Adolescent Health (DZKJ), partner site Berlin, Berlin, Germany; 6 Biochemistry & Structural Biology, Université Libre de Bruxelles (ULB), Brussels, Belgium; 7 Cluster of Excellence ImmunoPreCept, Charité - Universitätsmedizin Berlin, Berlin, Germany; 8 Department of Chemistry, Humboldt-Universität zu Berlin, Berlin, Germany

**Keywords:** Chemical modification, Chemical biology, Proteins

## Abstract

Nanobodies are emerging as attractive biopharmaceuticals due to their small size, stability and target specificity. However, their therapeutic use has largely been restricted to extracellular targets because of a lack of efficient delivery methods. This limitation is particularly relevant for diseases caused by dysfunctional intracellular proteins, such as cystic fibrosis. Here we show that cell-permeable nanobodies can modulate an intracellular disease-relevant target: the cystic fibrosis transmembrane conductance regulator (CFTR) chloride channel carrying the common F508del mutation. By combining a CFTR-binding nanobody with cell-penetrating peptides, we achieved intracellular delivery in cystic fibrosis bronchial epithelial cells. The delivered nanobody stabilizes misfolded F508del-CFTR, promotes its maturation and trafficking to the apical membrane and restores chloride channel activity. Moreover, the cell-permeable nanobody enhances the efficacy of approved CFTR modulator drug combination in primary airway epithelial cultures from patients with cystic fibrosis. These findings establish cell-permeable nanobodies as promising biopharmaceuticals for intracellular protein targeting and therapeutic modulation.

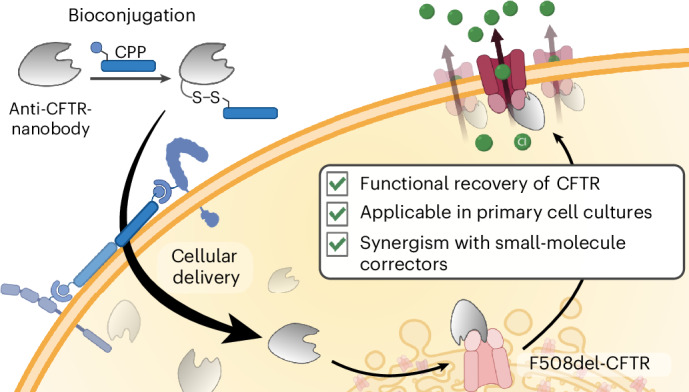

## Abstract (web summary)

Cell-permeable nanobodies overcome a major barrier to intracellular protein targeting. Franz, Rubil and colleagues show that delivery of a CFTR-binding nanobody into airway epithelial cells from patients with cystic fibrosis homozygous for the F508del-CFTR mutation stabilizes misfolded CFTR chloride channels and synergizes with the clinically approved modulator combination elexacaftor/tezacaftor/ivacaftor to restore CFTR function to near-normal levels.

## Main

Biopharmaceuticals are on the rise in pharmaceutical development, with monoclonal antibodies making up over half of the approved biopharmaceuticals in the United States and Europe between 2018 and 2022 (ref. ^1^). Although they are widely applied in treatment of cancer, inflammation and metabolic diseases, their application is limited by their inability for deep tissue penetration due to their size and immunogenicity^2^. Antibody fragments, such as single-chain variable fragments, as well as nanobodies, derived from the variable domain of heavy chain-only antibodies^3^, have received much attention to overcome these limitations. In particular, nanobodies pose several attractive features, including high solubility, high temperature, physicochemical stability^4,5^ and long shelf-life^6^. Large synthetic libraries for the selection and development of new targeted binders are available^7,8^. Moreover, nanobodies are straightforwardly accessible by expression in bacteria and can easily be modified by enzymatic or chemical modification strategies^4,7^. These aspects resulted in a wide array of applications in the biological and pharmacological sciences, which are further facilitated by their fast clearance, deep tissue penetration and low immunogenicity compared to conventional antibodies^9^.

Despite major advances in developing nanobodies for therapeutic applications, current nanobody modalities are limited to address extracellular targets due to a lack of cell-permeable variants. Nevertheless, it is well established that nanobodies are able to address intracellular targets to manipulate, modulate, inhibit or degrade relevant proteins in the form of intracellularly expressed nanobodies^7^. These so-called intrabodies rely on the delivery of genetic material into the cell to express the nanobody, which is suitable in cellulo but necessitates gene therapy in a clinical context. Additionally, intrabodies do not allow for non-genetic modification, which prevents enzymatic or chemical modifications for labeling or stabilization.

In recent years, several approaches have been pursued for the intracellular delivery of proteins and nanobodies, including physical methods to overcome the cell membrane by pore formation or membrane disruption^10–13^ as well as injection using a bacterial type III protein secretion system as a ‘molecular syringe’^14,15^. Furthermore, nanobody supercharging^16^ and the use of cyclic arginine-rich cell-penetrating peptides (CPPs)^17,18^ or cell-surface-anchoring CPP additives have been used^19^. However, proof-of-concept studies with cell-permeable nanobodies have thus far focused mainly on targeting green fluorescent protein (GFP) in engineered cell lines or on the labeling of endogenous protein targets, in particular for super-resolution microscopy^20^. Recently, the potential of nanobodies in cancer therapy was demonstrated by using a nanobody as an affinity binder in a targeted autophagic degradation approach^21^ and by using CPPs to create a cell-permeable nanobody that stops cancer proliferation by relocating and arresting function of a microtubule-associated protein in the cytosol^18^. However, no intracellular nanobody has been used yet to recover the function of an endogenous protein.

To demonstrate the prospect of cell-permeable nanobodies to modulate a therapeutically highly relevant intracellular target, we now present a cell-permeable nanobody, which reconstitutes the activity of the misfolded CFTR causing cystic fibrosis (CF). The most common mutation in patients with CF is a deletion of F508 in CFTR, which causes misfolding and intracellular degradation of CFTR anion channels, thus incapacitating insertion into the apical membrane, resulting in impaired transepithelial transport of chloride and bicarbonate, which are essential for host defense and homeostasis in the lungs and other epithelial organs^22–24^. Current therapeutic approaches focus on pharmacological rescue of F508del-CFTR using a combination of the small-molecule CFTR correctors elexacaftor and tezacaftor in combination with the potentiator ivacaftor (ETI)^25,26^. Although this triple combination therapy provides unprecedented improvement in clinical outcomes in patients with at least one copy of the common *F508del* mutation^27^, restoration of CFTR function remains partial, and chronic infection and inflammation of the lungs persist^28–31^, underscoring the need for further optimization of F508del correction^22^.

Previously, a CFTR-binding nanobody was shown to bind NBD1 of CFTR and thermally stabilize F508del-CFTR in vitro, suggesting its therapeutic potential to correct the folding defect and restore CFTR function^32–34^. Because small-molecule correctors bind F508del-CFTR outside of NBD1 and do not lead to thermal stabilization of F508del-CFTR^34,35^, the nanobody may represent a novel strategy to synergistically stabilize the mutant CFTR with approved modulators, making it a promising therapeutic candidate for CF^36,37^. However, therapeutic development of CFTR-targeting nanobodies has been hampered by the lack of tools for intracellular delivery, where CPPs could offer a technological advance to bridge this gap. In the present study, we selected a previously reported nanobody^32^ that exhibited high affinity against wild-type and F508del-CFTR to demonstrate the utility of cell-permeable nanobodies as tools to intracellularly modulate endogenous protein function. To render the nanobody cell permeable, we proposed to use our previously introduced CPP-additive strategy, which enabled the cytosolic delivery of protein−CPP conjugates at low concentrations^19,20^. We investigated the effects of intracellular nanobody delivery on maturation, trafficking and chloride channel function of F508del-CFTR in various cell lines and highly differentiated primary airway epithelial cultures from patients with CF. To assess these effects, we used a range of biochemical and functional methods, including confocal fluorescence live-cell microscopy, flow cytometry, western blot analysis and transepithelial short-circuit current (*I*_sc_) measurements.

## Results and Discussion

### Design of a cell-permeable F508del-CFTR-targeting nanobody

**Fig. 1:**
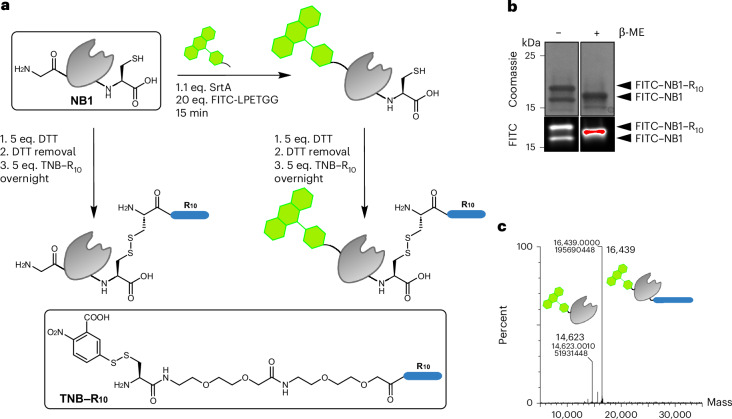
Design of a cell-permeable CFTR-binding nanobody. **a**, CPP conjugation and fluorescent labeling scheme. **b**,**c**, Analysis of fluorescent, cell-permeable nanobody NB1 (FITC−NB1−R_10_) by SDS-PAGE (**b**) and HR-MS (**c**), confirming successful sortase labeling and subsequent reducible CPP conjugation. The conjugation was replicated independently with similar results. min, minutes; SrtA, sortase A. Source data

We started our investigation by expressing a suitable cysteine-containing nanobody variant of a reported CFTR-binding nanobody^32^, further referred to as NB1, to allow the attachment of a CPP (deca-arginine (R_10_)) peptide via a disulfide. Thereby, the NB1−CPP conjugate can be taken up by the cell by direct transduction, and the disulfide-conjugated CPP can be cleaved in the intracellular reductive environment to ensure intracellular target engagement of the nanobody^17,38^. In addition, NB1 contains an N-terminal glycine after processing for sortase-mediated site-specific fluorophore labeling to track successful cellular uptake (Fig. 1a). Because the incorporation of a C-terminal cysteine can drastically reduce the yield of bacterial nanobody expression^39^, we optimized the expression and purification of NB1. Expression was performed in TB medium with additional glucose and Mg_2_Cl, to increase the protein yield. Furthermore, reductive conditions during all purification steps ensured a reduction of loss of protein due to dimerization. These changes resulted in a significant increase in yield compared to the previously established protocol for the unaltered nanobody variant^32^ (see Supplementary Fig. 1 for more information and Supplementary Fig. 2a,b for protein characterization data). N*-*terminal sortase labeling^40^ with 1.1 equivalents (eq.) of sortase and 20 eq. of fluorescein isothiocyanate (FITC)-conjugated LPETGG peptide and subsequent sortase removal yielded the desired fluorescent nanobody cysteine mutant (FITC−NB1). The final CPP conjugation was performed as previously described with 3 eq. of 5-thio-bis-(2-nitrobenzoic acid)−R_10_ (TNB−R_10_) overnight at 4 °C (ref. ^19^). The resulting cell-permeable fluorescent CPP-disulfide-conjugated nanobody (FITC−NB1−R_10_) was analyzed by SDS-PAGE (Fig. 1b) and high-resolution mass spectrometry (HR-MS) (Fig. 1c), confirming successful fluorescent labeling and CPP conjugation in 25% yield over two conjugation steps.

### Cytosolic delivery of a F508del-CFTR-targeting nanobody

**Extended Data Fig. 1:**
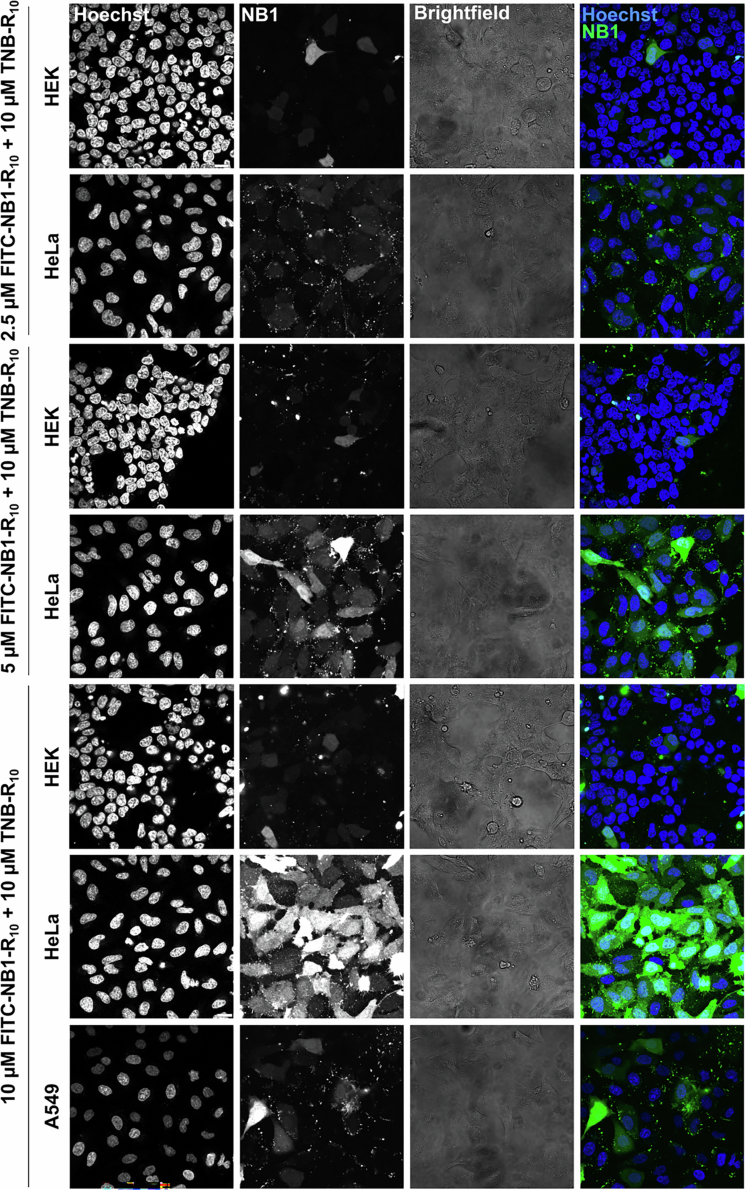
Cellular uptake of cell permeable NB1-R_10_ into different cell lines. HEK and A549 cells were incubated with 2.5 - 10 µM FITC-NB1-R_10_ (FITC) and 10 µM TNB-R_10_ in serum free FluoroBrite DMEM for 1 h at 37 °C, counterstained with Hoechst and imaged by confocal fluorescence live-cell microscopy in FluoroBrite DMEM with 10% FCS at RT. Scale bar: 20 µm.

For the delivery of the fluorescent CPP−nanobody conjugate FITC−NB1−R_10_, we used the thiol-reactive polyarginine-containing CPP (TNB−R_10_), used previously for CPP conjugation^19^, as an additive. First, we probed the delivery of FITC−NB1−R_10_ into different human cell lines (HeLa, HEK and A549). We observed cellular uptake and intracellular localization in endosomes, indicated by the punctuated signal, as well as in the cytosol, evident by diffuse fluorescence signal across the cytosol and nucleus of the cell in all cell lines at 10 µM FITC−NB1−R_10_/10 µM TNB−R_10_, (Extended Data Fig. 1)^41^. Because the nanobody needs to be available in the cytosol to carry out its estimated function, we investigated the required concentration threshold to observe successful cytosolic delivery in these cell lines. We observed uptake at nanobody/additive concentrations as low as 2.5 µM FITC−NB1−R_10_/10 µM TNB−R_10_ in HeLa and HEK cells, whereas successful cytosolic uptake in A549 cells was observed at 10 µM FITC−NB1−R_10_/10 µM TNB−R_10_.

**Fig. 2:**
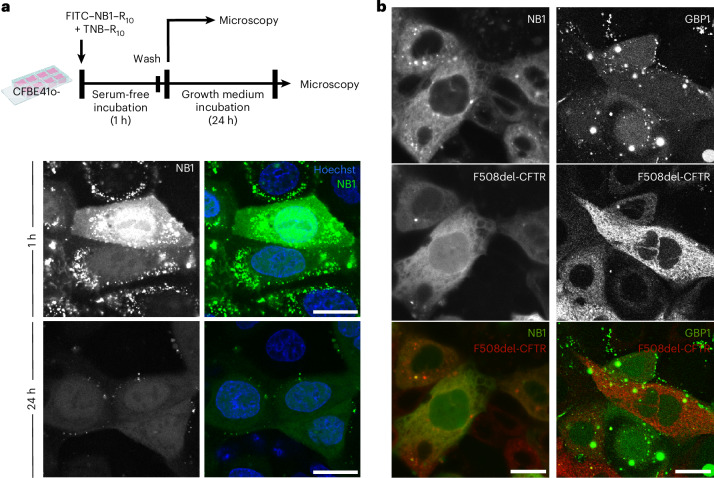
Cellular delivery of a CFTR-binding nanobody. **a**, Incubation scheme and confocal fluorescence live-cell microscopy images of cellular delivery in CFBE41o- cells with 10 µM FITC−NB1−R_10_/10 µM TNB−R_10_. Images were taken after either incubation with the nanobody for 1 hour or an additional 24-hour incubation in growth medium (25 hours). Scale bar, 20 µm. The uptake was replicated with similar results. **b**, Analysis of NB1 binding in cellulo by probing co-localization with CFTR. CFBE41o- cells expressing mCherry−Flag−F508del-CFTR were incubated for 1 hour with 10 µM FITC−NB1−R_10_/10 µM TNB−R_10_ (left column) or 10 µM FITC−GBP1−R_10_/10 µM TNB−R_10_ (right column) in serum-free medium followed by a 16-hour incubation in growth medium. Images were taken by confocal fluorescence live-cell microscopy. Representative images are shown; the experiment was replicated independently with similar results. Pearsonʼs correlation coefficients for co-localization were assessed with Fiji. Calculated Pearsonʼs correlation coefficient: *r*(NB1) = 0.65 ± 0.13 and *r*(GBP1) = 0.21 ± 0.10. Scale bar, 20 µm. h, hours.

Next, we tested the uptake in CF bronchial epithelial cells (CFBE41o-) expressing F508del-CFTR, a disease-relevant cell model of the CF airway epithelium suitable for functional studies^42,43^. CFBE41o- cells were treated for 1 hour in serum-free conditions and either directly subjected to confocal fluorescence live-cell imaging (1 hour) or further incubated for 24 hours in growth medium without nanobody prior to imaging (25 hours) (Fig. 2a). The uptake was first observed at 10 µM FITC−NB1−R_10_/10 µM TNB−R_10_, indicated by the clear fluorescence signal across the cytosol and the nucleus (Fig. 2a). Notably, a diminished but detectable cytosolic fluorescent signal remained 24 hours after nanobody removal, indicating sustained intracellular retention (Fig. 2a).

**Extended Data Fig. 2:**
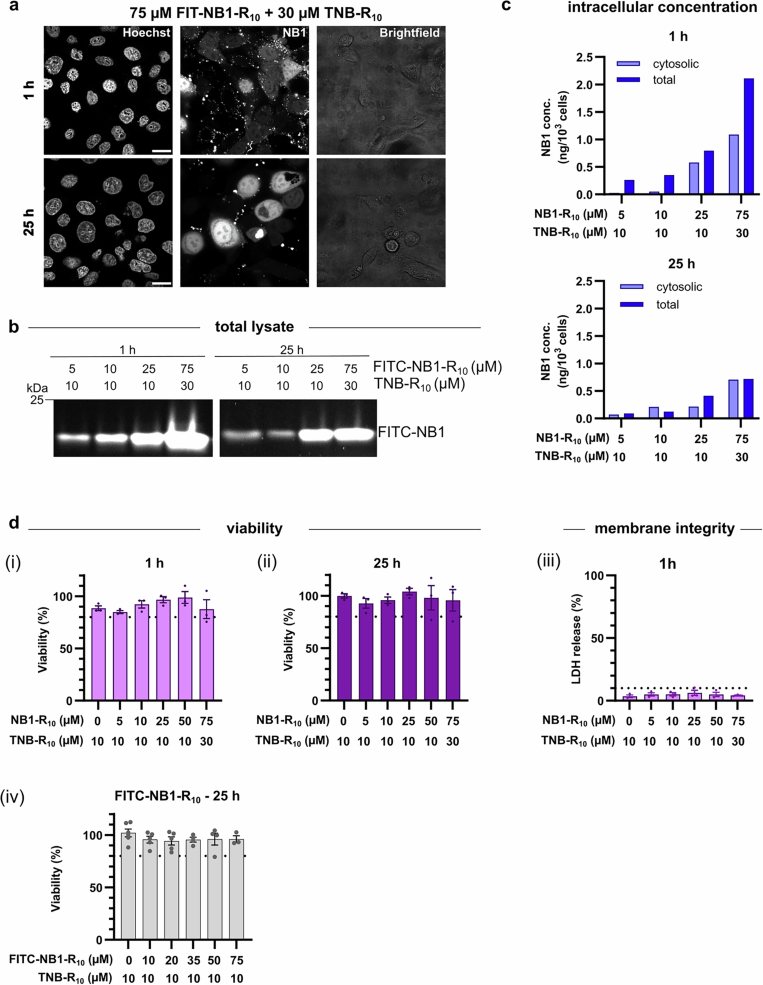
Analysis of NB1 delivery into CFBE41o- cells. **a** confocal fluorescence live-cell microscopy images of cellular delivery in CFBE41o- cells incubated with 75 µM FITC-NB1-R_10_/30 µM TNB-R_10_ for 1 h in serum-free medium (1 h) or after subsequent 24 h of incubation in growth medium (25 h). Scale bar: 20 µm. **b** NB1 concentration in lysed cells after incubation with indicated concentration of FITC-NB1-R10 and TNB-R10 directly after incubation (1 h) or after a subsequent 24 h incubation in growth medium (25 h) (full SDS PAGE gels see supplementary Fig. 4). The assay was replicated with similar results **c** Quantification of NB1 content in cell lysates (total) or cytosolic fraction of lysates (cytosolic). Cells were harvested and lysed after incubation (1 h) or 24 h post incubation (25 h) by Laemmli buffer (total) or lysed and fractioned to obtain cytosolic content (cytosolic). Nanobody amount was calculated by fluorescence intensity analysis of gel electrophoresis bands with the help of a concentration curve and normalized to the cell count of given sample. n = 1 distinct sample per condition **d** Cytotoxic effect of cellular delivery on CFBE41o- cells. Cell viability measured by WST-1 assay in CFBE41o- cells. Absorbance at 440 nm indicates cellular metabolic activity in the processing of WST-1 to the absorbing Formazan. Measured values were normalized to a medium treated control as 100% viability. Cells were treated with 0-75 µM NB1-R_10_ (i-ii) or FITC-NB1-R_10_ (iv) in presence of 10/30 µM TNB-R_10_ for 1 h and viability was directly determined (i) or subsequently incubated for 24 h prior to viability measurements (ii). Membrane integrity was determined by Lactase dehydrogenase (LDH) assay (iii). Emission at 590 nm indicates LDH release and membrane damage. Measured values were normalized to a trition X-100 treated control as maximal membrane damage and LDH release. Cells were treated with 0-75 µM NB1-R_10_ in presence of 10/30 µM TNB-R_10_ for 1 h and LDH content in medium was directly determined (1 h). Data presented as bars representing the mean ± SEM of n = 3 biological experiments (represented by dots) with 3 distinct samples per experiment (i-iii) or of n = 2 biolgoical experiments with n ≥ 2 distinct samples per experiment (represented by dots). Source data

Although incubation with 10 µM FITC−NB1−R_10_/10 µM TNB−R_10_ resulted in efficient cytosolic delivery, variability in uptake across the whole cell population was observed, ranging from predominantly endosomal uptake to clear cytosolic signals (Supplementary Fig. 3). To investigate if higher concentrations lead to a more uniform signal distribution, cells were incubated with 75 µM FITC−NB1−R_10_/30 µM TNB−R_10_. Qualitative imaging analysis revealed efficient cytosolic uptake after 1-hour incubation as well as clearly detectable intracellular signal persisting 24 hours after removal of nanobody (Extended Data Fig. 2a (1 hour and 25 hours)). Although a variance in fluorescence intensity across the population was still observed under these conditions, most of the cell population presented a cytosolic signal (Supplementary Fig. 3).

To quantify these imaging results and confirm that the observed fluorescent signal at 25 hours is not due to degradation and release of FITC, the cell lysate was analyzed by gel electrophoresis, and cellular protein content was quantified. Fluorescence analysis of the gel revealed a band at the expected molecular weight for all tested nanobody/additive concentrations (5−75 µM FITC−NB1−R_10_/10−30 µM TNB−R_10_) after 1-hour nanobody incubation (1 hour) as well as for 24 hours after nanobody removal (25 hours) (Extended Data Fig. 2b). To further investigate the amount of nanobody delivered to the cell, the fluorescence of the labeled and delivered nanobody was used. Cells were incubated for 1 hour with 5−75 µM FITC−NB1−R_10_/10−30 µM TNB−R_10_ in serum-free medium and collected either right after nanobody incubation (1 hour) or 24 hours after nanobody removal (25 hours). To obtain the total cellular protein content, samples were subsequently lysed with reducing Laemmli buffer. Alternatively, to obtain the cytosolic protein content, cytosolic fractioning was performed according to a previously published protocol^44^. After gel electrophoresis, fluorescence intensities of bands corresponding to NB1 were measured for each sample. Using an FITC−NB1 concentration curve, the protein amount per band was assessed (Extended Data Fig. 2c and Supplementary Fig. 4). As expected, after 1-hour incubation, NB1 can be detected in increasing concentrations corresponding to the applied nanobody concentration in both the total cellular content as well as the cytosolic fraction. Due to endosomes and nuclei being excluded from the cytosolic content, less nanobody was measured in the cytosolic fraction compared to the total cellular content. Twenty-five hours after initial nanobody application, the amount of nanobody detected in the cytosolic fraction and the total cellular content appear equilibrated, albeit lower relative to the amounts observed at 1 hour after nanobody application (Extended Data Fig. 2c). These observations confirm successful cytosolic delivery and demonstrate that a significant amount of nanobody was present in the cytosol of cells for at least 24 hours after removal of FITC−NB1−R_10_.

Next, we investigated the cell toxicity, delivery pathway and intracellular binding capacity of the cell-permeable nanobody. Because CPPs are known to be toxic for cells at high concentrations, we determined the membrane integrity and viability of cells. A lactate dehydrogenase (LDH) assay to determine membrane integrity after 1-hour NB1 incubation in serum-free conditions showed no significant increase in LDH release for all tested concentrations (0−50 µM NB1−R_10_/10 µM TNB−R_10_ and 75 µM NB1−R_10_/30 µM TNB−R_10_; Extended Data Fig. 2d (iii)). This indicates that the nanobody delivery, although most likely being carried out via direct translocation^19,45,46^ through the membrane, does not permanently damage the membrane to allow leakage of cellular content. Additionally, metabolic activity was assessed via a WST-1 assay to access cell viability after 1-hour incubation in serum-free media (1 hour) and after an additional 24-hour nanobody-free period in growth medium (25 hours). At both timepoints, all tested conditions (0−50 µM NB1−R_10_/10 µM TNB−R_10_ and 75 µM NB1−R_10_/30 µM TNB−R_10_) showed cell viabilities above 80−90% (Extended Data Fig. 2d (i)−(ii)). Conjugation of FITC to NB1−R_10_ did also not affect cell viability after 25 hours (Extended Data Fig. 2d (iv)). Taken together, these data show that the nanobody can be delivered to cells in concentrations up to 75 µM without cytotoxic or membrane-lytic effect, thus allowing to probe a wide concentration range to achieve functional recovery of F508del-CFTR.

Incubation with 10−50 µM FITC−NB1-R_10_/10 µM TNB−R_10_ at 4 °C demonstrated successful energy-independent cellular uptake, as previously demonstrated for other proteins^19,45^. Cells treated under these conditions showed diffuse fluorescent signals across the cytosol, indicating that direct translocation across the membrane takes place, as expected for cationic CPPs^47^ (Supplementary Fig. 5a). At 37 °C, we observed significant accumulation of fluorescent cell-permeable nanobodies in endosomes, as indicated by a co-localization with punctated signal with lysosomal tracker (Supplementary Fig. 5b). By performing a previously established endosome rupture assay^48^, we verified that no notable endosomal rupture occurs under these conditions compared to the positive control using the endosomolytic dipeptide LLOMe (Supplementary Fig. 5c). In light of these results, we assume that the main delivery mechanism of the nanobody in the cytosol does not occur via endosomal escape but, rather, direct transduction.

After the successful delivery of the nanobody into CFBE41o- cells, we performed co-localization experiments to determine whether the nanobody retained its CFTR binding capacity. Therefore, CFBE cells that express mCherry−Flag−F508del-CFTR upon induction were induced as previously described^49^ and treated with 10 µM FITC−NB1−R_10_/10 µM TNB−R_10_. Imaging was conducted 16 hours after incubation. In addition, cells were treated analogously with a cell-permeable FITC-conjugated and CPP-conjugated GFP-binding nanobody^50,51^ (FITC−GBP1−R_10_) to control for unspecific binding (Fig. 2b). Whereas uptake was observed for both nanobodies as indicated by intracellular fluorescent signals (Fig. 2b), only NB1 resulted in co-localization with the distinct CFTR−mCherry structures in the cell, whereas GBP1 is diffusely localized in the cytosol. This was corroborated by analysis of the Pearson’s correlation coefficient for 10−15 cells per treatment condition, indicating a co-localization for NB1 and CFTR (*r* = 0.65 ± 0.13), whereas no co-localization was determined for GBP1 and CFTR (*r* = 0.21 ± 0.10) (Supplementary Fig. 6). These data confirm the intracellular binding of the cell-permeable nanobody NB1 to F508del-CFTR in living cells, which was previously demonstrated in vitro as well as in permeabilized baby hamster kidney-21 cells stably expressing human CFTR by flow cytometry experiments^32^.

### Nanobody rescues F508del function in CFBE41o- cells

**Fig. 3:**
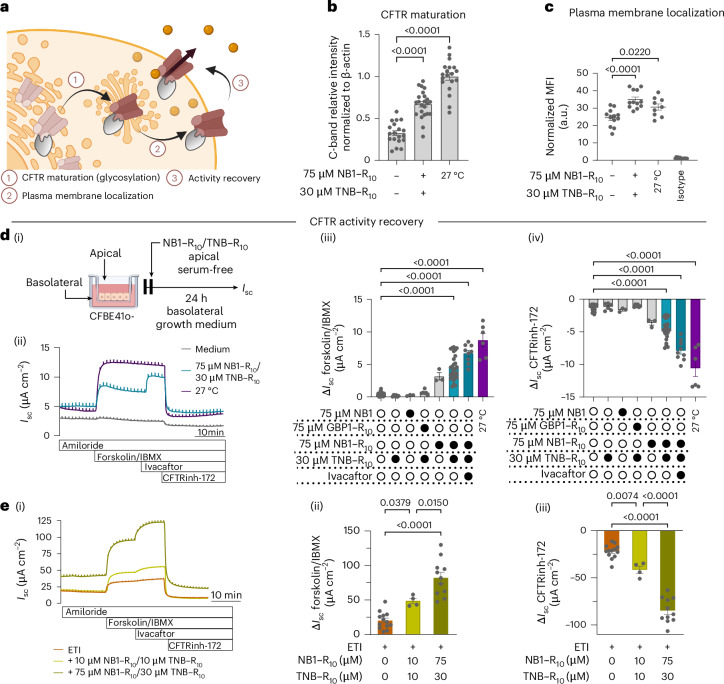
Nanobody treatment rescues F508del-CFTR maturation, trafficking and chloride channel function and enhances response to CFTR modulators in CFBE41o- cells. **a**, Schematic overview of nanobody-mediated rescue of F508del-CFTR trafficking and function. **b**, Analysis of CFTR maturation by immunoblot analysis of whole-cell lysates of CFBE41o- cells treated with vehicle control (medium) or 75 µM NB1−R_10_/30 µM TNB−R_10_ or low-temperature (27 °C) conditions. C-band intensities corresponding to mature, complex glycosylated CFTR were quantified and normalized to β-actin. Data were generated from *n* ≥ 4 independent samples. Bars represent mean ± s.e.m., with individual points representing technical replicates (*n* ≥ 19). Statistical significance was assessed by one-way ANOVA with Dunnett’s post hoc test using vehicle as control. Both NB1−R_10_/TNB−R_10_ and 27 °C treatments differed significantly compared to vehicle (*P* < 0.0001). **c**, Analysis of CFTR restoration via CFTR cell surface content determined in CFBE41o- cells. Cells were treated with 75 µM FITC−NB1−R_10_/30 µM TNB−R_10_, and controls were incubated with medium at 37 °C (medium) or 27 °C (positive control). Subsequent to CFTR antibody staining of non-permeabilized cells (CFTR monoclonal antibody (CF3) (Invitrogen) and goat anti-mouse IgM (heavy chain) secondary antibody, Alexa Fluor 647)), CFTR cell surface content was quantified by flow cytometry. As antibody staining control, untreated samples were treated with an isotype control antibody (isotype staining). Data are presented as a bar graph representing mean ± s.e.m. of *n* ≥ 12 distinct samples across three biological independent experiments (represented by dots). Statistical significance was determined by one-way ANOVA with Dunnett’s post hoc test; NB1−R_10_/TNB−R_10_ and 27 °C were significantly different from control (medium) (NB1−_10_/TNB−R_10_ *P* < 0.0001; 27 °C *P* = 0.0220). **d**, Rescue of F508del-CFTR-mediated chloride transport by NB1−R_10_/TNB−R_10_. (i) Incubation scheme for Ussing chamber experiments. (ii) Representative original recordings of *I*_sc_ measurements in CFBE41o- monolayers treated with vehicle alone, 75 μM NB1−R_10_/30 μM TNB−R_10_ in combination with CFTR potentiator ivacaftor or low temperature (27 °C) for 24 hours. (iii) Quantification of forskolin/IBMX-induced short-circuit current (Δ*I*_sc_) and (iv) CFTRinh-172-sensitive Δ*I*_sc_ under the indicated treatment conditions. Treatments included unconjugated nanobody (NB1, 75 μM), CPP-conjugated nanobody (NB1−R_10_, 75 μM), CPP additive (TNB−R_10_, 30 μM) and a non-specific, GFP-binding nanobody (GBP1−R_10_, 75 μM) as well as combination treatments; vehicle-treated cells served as controls. Bars represent mean ± s.e.m.; individual points represent independent measurements from individual inserts. For forskolin/IBMX-induced Δ*I*_sc_: *n* = 25 (vehicle); *n* = 18 (NB1−R_10_/TNB−R_10_); *n* = 6 (TNB−R_10_, NB1, GBP1−R_10_/TNB−R_10_, 27 °C); *n* = 3 (NB1−R_10_); *n* = 9 (NB1−R_10_/TNB−R_10_/ivacaftor). For CFTRinh-172-sensitive Δ*I*_sc_: *n* = 18 (vehicle, NB1−R_10_/TNB−R_10_); *n* = 6 (TNB−R_10_, NB1, GBP1−R_10_/TNB−R_10_, 27 °C); *n* = 3 (NB1−R_10_); *n* = 9 (NB1−R_10_/TNB−R_10_/ivacaftor). Statistical significance was assessed by one-way ANOVA followed by Dunnett’s post hoc test using vehicle as the control. NB1−R_10_/TNB−R_10_, NB1−R_10_/TNB−R_10_/ivacaftor and 27 °C differed significantly from vehicle (*P* < 0.0001). **e**, Rescue of F508del-CFTR-mediated chloride transport in CFBE41o- monolayers treated with ETI alone or co-treated with ETI and different concentrations of NB1−R_10_/TNB−R_10_. (i) Representative original recordings of *I*_sc_ measurements. (ii) Quantification of forskolin/IBMX-induced Δ*I*_sc_ and (iii) CFTRinh-172-sensitive Δ*I*_sc_. Bars represent mean ± s.e.m. Individual points represent independent measurements from individual inserts: *n* = 13 (ETI); *n* = 4 (ETI/NB1−R_10_/TNB−R_10_, 10 µM/10 µM); *n* = 11 (ETI/ NB1−R_10_/TNB−R_10_, 75 µM/30 µM). Statistical significance was assessed by one-way ANOVA with Tukey’s multiple comparisons test. The exact *P* values are reflected in the figure. Panel **a** created in BioRender; Franz, L. https://biorender.com/2gk7i0f (2026). h, hours; min, minutes. Source data

**Extended Data Fig. 3:**
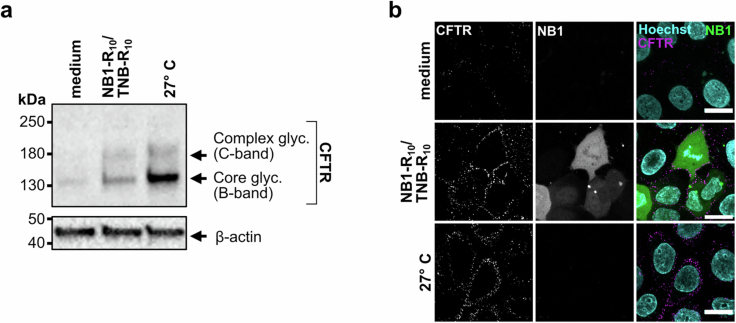
CFTR maturation and trafficking analysis. **a** Analysis of CFTR maturation by immunoblot analysis of whole cell lysates of CFBE41o- cells treated with vehicle control (medium) or 75 µM NB1-R_10_/30 µM TNB-R_10_, or low temperature (27 °C) conditions. Representative CFTR Western blot image showing CFTR B-band (core glycosylated CFTR) and CFTR C-band (complex glycosylated CFTR) with β-actin as a loading control. **b** Analysis of CFTR restoration via CFTR cell surface content determined in CFBE41o- cells by confocal fluorescence live-cell microscopy. Cells were treated with 75 µM FITC-NB1-R_10_/30 µM TNB-R_10_ or incubated with medium at 37 °C (medium) or 27 °C as positive control. Afterwards, cells were analyzed subsequent to CFTR antibody staining (CFTR Monoclonal Antibody (CF3) (Invitrogen) and goat anti-mouse IgM (heavy chain) secondary antibody, Alexa Fluor™ 647, top row: cells treated for 1 h with 75 µM FITC-NB1-R_10_/30 µM TNB-R_10_ in serum-free Fluorobrite DMEM and subsequently incubated for 16 h in growth medium. Middle row: cells treated for 1 h in serum-free Fluorobrite DMEM (medium) and subsequently incubated for 16 h in growth medium. Bottom row: Cells incubated continuously in growth medium for 17 h at 27 °C for temperature correction. Scale bar: 20 µm. The experiment was replicated with similar results Source data

Based on these results, we investigated if a non-fluorescent, cell-permeable nanobody (NB1−R_10_) could intracellularly stabilize misfolded F508del-CFTR and restore CFTR-mediated chloride transport in CFBE41o- cells after cellular delivery. NB1−R_10_ was obtained by CPP conjugation of the purified NB1 (scheme in Fig. 1a; for protein characterization data, see Supplementary Fig. 2c,d). We investigated the effect of the cell-permeable nanobody NB1−R_10_ on the maturation and plasma membrane trafficking of F508del-CFTR (Fig. 3a). We first evaluated CFTR maturation upon cell-permeable nanobody treatment by western blot analysis. CFBE41o- cells were cultured as confluent monolayers on semi-permeable filters and treated apically with 75 µM NB1−R_10_/30 µM TNB−R_10_ for 24 hours in serum-free medium. As positive control, CFBE41o- cells were incubated at low temperature (27 °C) for 24 hours, which was previously shown to restore folding and function of F508del-CFTR^52^. We observed two distinct bands in both nanobody-treated and temperature-corrected (27 °C) groups that correspond to the core glycosylated (B-band) and complex glycosylated (C-band) form of CFTR (Extended Data Fig. 3a), whereas the untreated cell lysates showed only the presence of the B-band^25,52^. Densitometric analysis confirmed that the relative amount of CFTR C-band protein was significantly increased in both NB1−R_10_ and 27 °C groups compared to untreated controls (Fig. 3b), demonstrating increased CFTR maturation associated with increased trafficking from the endoplasmic reticulum to the Golgi apparatus.

Next, we determined whether the maturation of F508del-CFTR upon nanobody treatment improves trafficking of the corrected CFTR to the apical membrane and results in increased CFTR cell surface levels in CFBE41o- cells. Therefore, CFBE41o- cells were incubated for 1 hour with 75 µM FITC−NB1−R_10_/30 µM TNB−R_10_ in serum-free medium with a subsequent 16-hour incubation in growth medium to allow for maturation and relocation of F508del-CFTR to the cell surface (Fig. 3c and Extended Data Fig. 3b). Again, low-temperature correction (27 °C) was performed for 17 hours as control^52^. To evaluate cell surface CFTR, live cells were stained with an antibody against an extracellular loop peptide sequence of CFTR and subsequently either analyzed by microscopy (Extended Data Fig. 3b and Supplementary Fig. 7a) or quantified by flow cytometry analysis (Fig. 3c). Confocal fluorescence microscopy revealed that a CFTR signal could be observed only in temperature-corrected and nanobody-treated CFBE41o- cells but not in CFBE41o- cells treated with medium alone, indicating an increase in cell surface CFTR in these conditions. For flow cytometry measurements, single, intact cells were gated and analyzed. In case of FITC−NB1−R_10_-treated samples, only the FITC-positive population (cells containing NB1) was considered in the analysis (Supplementary Fig. 7b). The allophycocyanin (APC) channel mean fluorescence intensity (MFI) was analyzed for the appropriate populations, as it is considered to correlate with CFTR levels on the cell surface in a given sample. In accordance with the microscopy results, a higher normalized MFI was detected for nanobody-treated cells as well as temperature-corrected cells compared to the untreated controls, indicating a higher abundance of CFTR on the cell surface in these treatment groups (Fig. 3c). Taken together, these data show that the intracellular delivery of NB1−R_10_ corrects the folding and maturation of F508del-CFTR and subsequently allows its trafficking to the plasma membrane in CFBE41o- cells.

**Extended Data Fig. 4:**
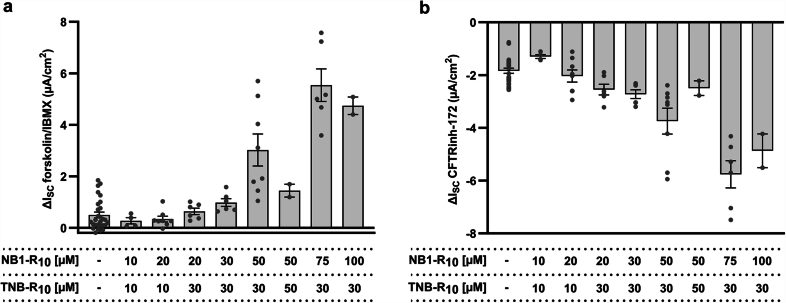
Dose response study. **a**-**b** Initial dose-response study to evaluate the effects of treatment with different concentrations of NB1-R10 (0-100 μM) in combination with CPP additive (TNB-R10, 10-50 μM) on forskolin/IBMX-induced ΔIsc (**a**) and CFTRinh-172-sensitive ΔIsc (**b**) in F508del-CFTR expressing CFBE41o- cells. Bars show ± SEM. Individual points represent independent measurements from individual inserts: n = 28 (vehicle); n = 4 (10 µM/10 µM), n = 8 (20 µM/10 µM), n = 6 (20 µM/30 µM), n = 6 (30 µM/30 µM), n = 8 (50 µM/30 µM), n = 2 (50 µM/50 µM), n = 6 (75 µM/30 µM) n = 2 (100 µM/30 µM). Source data

To assess functional rescue of F508del-CFTR-mediated chloride transport, we conducted dose−response studies by *I*_sc_ measurements in Ussing chambers. CFBE41o- cells were cultured as a confluent monolayer on semi-permeable filters and incubated apically for 24 hours with NB1−R_10_ and TNB−R_10_ additives in serum-free medium. Rescue of F508del-CFTR function by NB1−R_10_/TNB−R_10_ was assessed by measuring changes in *I*_sc_ in response to cAMP-dependent activation with forskolin/IBMX and inhibition with CFTR inhibitor-172 (CFTRinh-172). Dose−response studies revealed a dose-dependent increase of CFTR function in CFBE41o- monolayers, with a maximal effect observed at 75 µM NB1−R_10_/30 µM TNB−R_10_, as evidenced by substantial increases in forskolin/IBMX-induced and CFTRinh-172-sensitive *I*_sc_. Increasing the concentrations of nanobody or additive beyond these levels did not lead to additional improvements in CFTR function (Extended Data Fig. 4a,b and Supplementary Fig. 8). This corresponds well with the increase in intracellular nanobody content observed with increasing applied protein concentrations (Extended Data Fig. 2c). Based on the cytosolic concentration determination (Extended Data Fig. 2c), we can assume that the average cell treated with 75 µM NB1−R_10_/30 µM TNB−R_10_ contains a cytosolic nanobody concentration of approximately 23 µM nanobody at the timepoint of measurement (assuming an average cell volume of 2 pl). Although a slight recovery of CFTR activity is already detectable at lower treatment conditions (30 µM NB1−R_10_/30 µM TNB−R_10_) by *I*_sc_ measurements, there is a discrepancy between the intracellular nanobody concentration necessary for CFTR activity rescue and the in vitro determined binding affinity (*K*_d_ = 54 ± 10 nM (ref.^32^). Similar disparities of binding affinities in protein−protein interactions determined under in vitro conditions and within the cellular context were reported and investigated previously^53–56^. Multiple factors could explain these differences. Macromolecular crowding and compartmentalization are not found under in vitro conditions, and the cellular distribution of the nanobody upon delivery could impact accessibility and binding ability of the nanobody to CFTR, resulting in higher applied concentrations being required^54^. Additionally, the three-dimensional structure and accessibility of F508del-CFTR and, specifically, its targeted binding site might differ when embedded into the endoplasmic reticulum membrane after expression compared to the isolated protein(-domain) used in in vitro studies. Although the binding site is still accessible from the cytosol, the changes in structure of the instable F508del-CFTR after expression and its constraint to the endoplasmic reticulum compartment might change the binding capacity of the nanobody in cellulo^54^. Structural changes of the nanobody based on the reducing intracellular environment compared to in vitro conditions are not expected, because a stable structure including disulfide bonds was previously obtained under reducing conditions (1 mM TCEP)^32^. To further evaluate and address the impact of each of these factors, a detailed, systematic study of in cellulo binding as well as the exact recovery mechanism is necessary.

To further evaluate to what extent the NB1-dependent correction of maturation and folding of F508del-CFTR translates to the recovery of its chloride channel function in CFBE41o- cells, we monitored CFTR activity under different experimental conditions, including incubation of the unconjugated CFTR-nanobody (NB1), incubation without the CPP additive (TNB−R_10_), incubation with the TNB−R_10_ alone and incubation of an unspecific CPP−nanobody conjugate (GBP1−R_10_) (Fig. 3e(i)). As positive control, low-temperature correction (27 °C) was performed^52^. In a subset of experiments, the CFTR potentiator ivacaftor was added after stimulation with forskolin/IBMX. The *I*_sc_ measurements in treated CFBE41o- monolayers showed that incubation with NB1 or TNB−R_10_ alone, or a cell-permeable GFP-binding nanobody^17,51^ (GBP1−R_10_) prepared in the same way as NB1, failed to increase forskolin/IBMX-induced or CFTRinh-172-sensitive *I*_sc_, confirming that the restoration of CFTR function was mediated by cytosolic delivery of NB1−R_10_ (Fig. 3d(iii−iv)). We observed that incubation with 75 µM NB1−R_10_ without TNB−R_10_ additive already showed an increase in CFTR-mediated chloride current—however, not to the level measured after co-treatment with TNB−R_10_ (Fig. 3d(iii−iv)). This can be expected, as cytosolic delivery of CPP-conjugated proteins can occur at higher concentrations^57^. Interestingly, in the presence of forskolin/IBMX, CFTR-mediated currents were further increased by applying the CFTR potentiator ivacaftor^58^ to NB1−R_10_/TNB−R_10_-treated CFBE41o- cells. A synergistic effect of the CFTR potentiator ivacaftor has been widely demonstrated when used in combination with small-molecule CFTR correctors, such as lumacaftor, elexacaftor and tezacaftor^25,26,59^. Of note, in combination with ivacaftor, the correction of F508del-CFTR chloride channel function achieved by NB1−R_10_ treatment reached levels similar to those of low-temperature correction (Fig. 3d(ii−iv)), which was previously shown to rescue F508del-CFTR folding and function^52^.

The current standard of care for patients with CF who carry at least one *F508del* allele is a triple combination CFTR modulator therapy consisting of the CFTR folding correctors elexacaftor and tezacaftor along with the potentiator ivacaftor (elexacaftor/tezacaftor/ivacaftor (ETI))^22,28^. In addition to assessing the effect of co-treatment with CPP−nanobody and the potentiator ivacaftor, we also investigated the impact of co-treatment with CPP−NB1 and ETI on the rescue of F508del-CFTR-mediated chloride channel function. To this end, confluent monolayers of CFBE41o- cells were co-treated with either 10 µM NB1−R_10_/10 µM TNB−R_10_ or 75 µM NB1−R_10_/30 µM TNB−R_10_ added to the apical side and elexacaftor and tezacaftor added to the basolateral medium for 24 hours. Ivacaftor was added acutely during the *I*_sc_ measurements, and CFTR function was determined by measuring changes in forskolin/IBMX-induced and CFTRinh-172-sensitive *I*_sc_ (Fig. 3e(i−iii)). Treatment of CFBE41o- monolayers with ETI alone induced a robust CFTR-mediated *I*_sc_ (Fig. 3e). Co-treatment of ETI with CPP−NB1 dose-dependently increased the restoration of F508del-CFTR chloride channel function in CFBE41o- cells. 10 µM NB1−R_10_/10 µM TNB−R_10_ increased the ETI-mediated rescue of CFTR function, as determined from the CFTRinh-172-sensitive *I*_sc_, by approximately 1.8-fold, which was further increased to approximately 3.8-fold by 75 µM NB1−R_10_/30 µM TNB−R_10._ This finding suggests a significant synergistic effect of NB1 and CFTR modulators. Control experiments with unspecific CPP−nanobody conjugate (GBP1−R_10_) demonstrated that co-treatment with 75 µM GBP1−R_10_/30 µM TNB−R_10_ and ETI enhanced neither forskolin/IBMX-induced *I*_sc_ nor CFTRinh-172-sensitive *I*_sc_ compared to ETI alone, further supporting the specificity of NB1 to rescue F508del-CFTR function (Supplementary Fig. 9). These results support that targeting the F508del mutation-containing NBD1 with cell-permeable NB1 acts synergistically with small-molecule CFTR correctors that bind in the transmembrane regions. This synergistic action enhances the efficacy on functional CFTR rescue, reaching levels that surpass those predicted by the sum of their individual effects^22,32,35^. Taken together, our findings support that improvement of NBD1 folding and stability of F508del-CFTR by the nanobody and global conformational protein stabilization by small-molecule CFTR correctors are complementary mechanisms that enhance efficacy of restoration of F508del chloride channel function, highlighting the therapeutic potential of the nanobody as an add-on therapy in CF^33–37,60^.

### Nanobody rescues F508del-CFTR function in primary CF airway epithelia

**Fig. 4:**
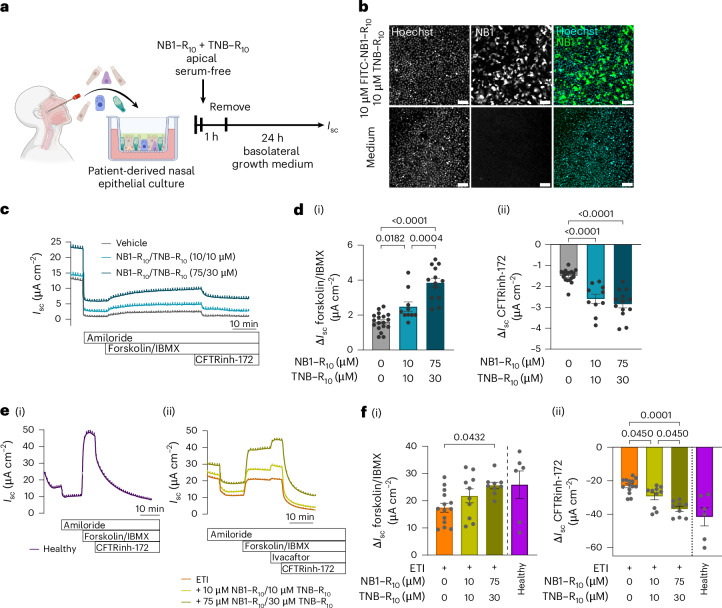
Nanobody treatment rescues F508del-CFTR chloride channel function and enhances response to CFTR modulators in CF primary airway cultures. **a**, Schematic illustration of generating highly differentiated airway epithelial cultures derived from nasal brushings of three healthy individuals and three patients with CF. **b**, Representative confocal images of airway epithelial cultures from a patient with CF homozygous for F508del-CFTR treated with either vehicle or FITC-labeled NB1−R_10_/TNB−R_10_ (10 μM/10 μM) and counterstained with Hoechst for live-cell imaging. Experiments were repeated independently four times with similar results. Cyan: Hoechst (cell nuclei); green: FITC. Scale bar, 50 μm. **c**, Representative original recordings of Ussing chamber measurements of *I*_sc_ in CF primary airway epithelial cultures treated with vehicle or with different concentrations of NB1−R_10_/TNB−R_10_. **d**, (i) Quantification of forskolin/IBMX-induced Δ*I*_sc_ and (ii) CFTRinh-172-sensitive Δ*I*_sc_. Bars show mean ± s.e.m. Individual points represent independent measurements from individual inserts: *n* = 18 (vehicle); *n* = 10 (NB1−R_10_/TNB−R_10_, 10 µM/10 µM); *n* = 13 (NB1−R_10_/TNB−R_10_, 75 µM/30 µM). Statistical significance was assessed by one-way ANOVA with Tukey’s post hoc test. For forskolin/IBMX-induced Δ*I*_sc_, vehicle differed from NB1−R_10_/TNB−R_10_ (10 μM/10 μM; *P* < 0.0182) and (75 μM/30 μM; *P* < 0.0001)), and the two concentrations differed from each other (*P* = 0.0004). For CFTRinh-172-sensitive Δ*I*_sc_, vehicle differed from NB1−R_10_/TNB−R_10_ (10 μM/10 μM) and (75 μM/30 μM)) (*P* < 0.0001 for both). **e**, (i) Representative original recordings of *I*_sc_ measurements in airway epithelial cultures derived from a healthy individual; (ii) representative original recordings of *I*_sc_ in CF airway epithelial cultures treated with ETI alone or co-treated with ETI and different concentrations of NB1−R_10_/TNB−R_10_. **f**, (i) Quantification of forskolin/IBMX-induced Δ*I*_sc_ and (ii) CFTRinh-172-sensitive Δ*I*_sc_. Bars show mean ± s.e.m.; individual points represent independent measurements from individual inserts: *n* = 14 (ETI); *n* = 10 (ETI/NB1−R_10_/TNB−R_10_, 10 µM/10 µM); *n* = 9 (ETI/NB1−R_10_/TNB−R_10_, 75 µM/30 µM). Statistical significance was assessed by one-way ANOVA with Tukey’s post hoc test. For forskolin/IBMX-induced Δ*I*_sc_, NB1−R_10_/TNB−R_10_/ETI (75 μM/30 μM) differed from ETI alone (*P* = 0.0432). For CFTRinh-172-sensitive Δ*I*_sc_, NB1−R_10_/TNB−R_10_/ETI (10 μM/10 μM) and (75 μM/30 μM) differed from ETI (*P* = 0.0450 and *P* < 0.0001, respectively), and the two concentrations differed from each other (*P* = 0.0450). Panel **a** created in BioRender; Franz, L. https://biorender.com/qizzos4 (2026). h, hours; min, minutes. Source data

Building on previous results demonstrating functional recovery of F508del-CFTR upon treatment with the cell-permeable nanobody in CFBE41o- cells, we investigated whether this effect can also be achieved in patient-derived primary airway epithelial cultures as a translational model that has been instrumental for the development of CFTR modulator drugs. To this end, we generated highly differentiated primary nasal epithelial cultures from patients with CF homozygous for the *F508del* allele^61^ (Fig. 4a). Although singular examples of cationic CPPs being used for therapeutically relevant protein delivery in primary cells exist^62^, human primary cells, especially airway epithelial cells, are generally known to be difficult to transfect^63,64^. Furthermore, patient-derived airway cultures recapitulate the mucus barrier, which needs to be penetrated by inhaled therapies for efficient pulmonary drug delivery^65^. Therefore, we initially tested the delivery of FITC−NB1−R_10_ into patient-derived cells. Patient-derived nasal epithelial cells were treated by apical application of 10 µM FITC−NB1−R_10_/10 µM TNB−R_10_ for 3 hours in PBS and subsequently imaged by confocal fluorescence live-cell microscopy 24 hours after treatment initiation. A diffuse signal across a substantial number of cells was observed, indicating successful cytosolic delivery (Fig. 4b). Next, we investigated the effect of the nanobody treatment on the restoration of F508del-CFTR chloride channel function. Similarly, patient-derived nasal epithelial cultures were treated with 10 µM NB1−R_10_/10 µM TNB−R_10_ or 75 µM NB1−R_10_/30 µM TNB−R_10_, and CFTR function was determined by *I*_sc_ measurements as described above (Fig. 4c,d). Consistent with live-cell imaging data suggesting efficient delivery of low-concentration NB1−R_10_/TNB−R_10_ into patient-derived cells, we observed a significant increase in forskolin/IBMX-induced and CFTRinh-172-sensitive *I*_sc_ after treatment with both 10 µM NB1−R_10_/10 µM TNB−R_10_ and 75 µM NB1−R_10_/30 µM TNB−R_10_. Taken together, these findings demonstrate successful delivery of CPP-conjugated nanobody in highly differentiated CF primary airway epithelial cells, resulting in partial restoration of F508del-CFTR function even at lower nanobody−CPP concentrations. Next, we investigated the synergistic potential of the nanobody when combined with approved CFTR correctors elexacaftor and tezacaftor in patient-derived airway cultures (Fig. 4e,f). To benchmark our experiments to wild-type CFTR function, we measured *I*_sc_ in primary nasal epithelial cultures from three healthy individuals (Fig. 4e(i),f). Consistent with previous studies^28,66^, ETI-mediated restoration of CFTR function, as determined by the ratio of the mean CFTRinh-172-sensitive *I*_sc_, was approximately 55% of that observed in healthy cultures. Similar to the experiments in the CFBE41o- cell line, co-treatment of the nanobody with ETI further enhanced functional rescue of CFTR function in CF nasal cultures. Specifically, the add-on treatment with 10 µM NB1−R_10_/10 µM TNB−R_10_ increased CFTR-mediated chloride secretion in CF cultures to approximately 70% of that in healthy cultures (Fig. 4e,f). Functional rescue of F508del-mediated chloride secretion was further enhanced by co-treatment of ETI with 75 µM NB1−R_10_/30 µM TNB−R_10_, leading to approximately 89% of CFTR function in healthy cultures (Fig. 4e,f). Overall, these data underscore the potential of cationic CPPs to overcome the challenge of delivering biotherapeutics into difficult-to-transfect primary cells and support the capacity of the cell-permeable CFTR-binding nanobody to further enhance rescue of F508del-CFTR function that is currently achieved with approved CFTR modulators up to near-normal levels. Therefore, this approach represents a novel promising strategy for improving the treatment of patients with CF with at least one *F508del* allele.

In conclusion, our data demonstrate that the CPP-mediated delivery of a functional CFTR-binding nanobody can modulate the fate of misfolded intracellular F508del-CFTR to restore its physiological function in a translational model of CF. By an array of biochemical and functional assays including fluorescent microscopy, flow cytometry and *I*_sc_ measurements, we show that the cell-permeable nanobody binds intracellularly to F508del-CFTR and corrects its folding defect to facilitate its maturation and trafficking. Ultimately, the cell-permeable nanobody rescues the function of F508del-CFTR chloride channel at the apical plasma membrane. To our knowledge for the first time, we successfully applied CPP-mediated protein delivery in primary human cells and demonstrated effective rescue of F508del-CFTR function by a nanobody in CF patient-derived primary airway epithelial cultures. Furthermore, we demonstrate that by targeting the NBD1 folding defect of F508del-CFTR, the nanobody exerts a synergistic effect when combined with the clinically approved triple combination CFTR modulator therapy ETI that includes two small-molecule correctors that bind outside NBD1 and stabilize the F508del protein, thereby improving its maturation and trafficking to the apical cell membrane. Of note, this combination achieved near-normal levels of CFTR function in primary airway cultures from patients with CF, highlighting the potential of nanobody-based therapies to address the limitations of partial functional correction achieved by currently available CFTR modulator drugs.

Using the CF-causing mutation *F508del* in the CFTR protein as an example, this study highlights the potential of intracellular applications of extracellularly applied nanobodies for therapeutic modulation of intracellular disease mechanisms such as protein misfolding in CF and showcases the CPP-additive technology as a powerful delivery tool for that purpose not only in cell lines but also in until-now-neglected primary cells. To develop cell-permeable nanobodies as inhaled therapies to further optimize pharmacological rescue of F508del-CFTR toward full correction, future studies will need to focus on optimizing mucus penetration, to overcome an additional substantial barrier in the lungs of patients with CF^22^. In this context, potential formulations need to be studied that allow the cell-permeable nanobody as well as the CPP additive to reach the cells in the airway lumen. Beyond CF, intracellular application of nanobodies may be a promising therapeutic approach to restore protein folding and function in many other rare genetic diseases with high unmet medical need.

## Methods

### General

Solvents (DMF and CH_2_Cl_2_) were purchased from Thermo Fisher Scientific. Amino acids, Rink amide resin and coupling reagents were purchased from Iris Biotech. HATU was purchased from Bachem. DIEA and TFA were purchased from Carl Roth. Salts, LB medium, antibiotics and other buffer components were purchased from Carl Roth. Mammalian cell culture media and FBS were purchased from VWR. Ultra-high-performance liquid chromatography-ultraviolet (UPLC-UV) traces were obtained on a Waters H-class instrument equipped with a Quaternary Solvent Manager, a Waters autosampler and a Waters TUV detector with an Acquity UPLC-BEH C18 1.7-μm, 2.1 × 50-mm RP column. Empower 3 software (Waters) was used.

Preparative high-performance liquid chromatography (HPLC) of peptides was done on a Gilson PLC 2020 system using a Nucleodur C18 Htec Spum column (Macherey-Nagel, 100 Å, 5 m, 250 mm × 32 mm, 30 ml min^−1^). The following gradient was used in all purifications: A = water + 0.1% TFA, B = MeCN + 0.1% TFA 5% B 0−10 minutes, 5−50% B 10−60 minutes, 50−99% 60−80 minutes. High-resolution mass spectra were measured on a Xevo G2-XS QTof (Waters) mass spectrometer coupled to an acquity UPLC system running on water and acetonitrile, both with 0.01% formic acid using MassLynx software (version 4.1, Waters). Protein spectra were deconvoluted using the MaxEnt 1 tool. For SDS-PAGE analysis, proteins were mixed with 4× Laemmli buffer (Bio-Rad) with or without addition of 10% β-mercaptoethanol (β-ME) and boiled at 95 °C for 5 minutes before separation on 4–20% Mini-PROTEAN TGX Precast Protein Gels (Bio-Rad). In-gel fluorescence was imaged first, followed by Coomassie staining and imaging. Gels were imaged on a ChemiDoc XRS+ system (Bio-Rad) using Image Lab software (version 5.1, Bio-Rad).

### Peptide synthesis

The TNB−R_10_ (ac-C(TNB)-PEG-PEG-RRRRRRRRRR, PEG: 8-amino-3,6-dioxaoctanoic acid, A) and FITC-LPETGG (B) peptides were synthesized by (automated) standard fluorenylmethoxycarbonyl (Fmoc) solid-phase peptide synthesis (SPPS) on Rink amide resin (0.05 mmol scale, 0.22 mmol g^−1^). Arginine was incorporated with Pbf protection; cysteine was incorporated on the N terminus with S-tbutyl protection. Selective deprotection of Fmoc-protected resin and Fmoc-protected amino acids was achieved with 20% piperidine in DMF. Amino acid coupling was performed with 5 eq. of amino acid, 5 eq. of *O*-(1H-6-chlorobenzotriazole-1-yl)-1,1,3,3-tetramethyluronium hexafluorophosphate (HCTU), 4 eq. of ethyl cyanohydroxyiminoacetate (Oxyma) and 10 eq. of *N*,*N*-diisopropylethylamine (DIEA) in DMF. The FITC coupling was done on the N terminus after Fmoc deprotection using 2 eq. of FITC (5(6)-carboxyfluorescein; Fluka, 21877), 2 eq. of hydroxybenzotriazole (HOBt) and 2 eq. of *N*,*N*′-diisopropylcarbodiimide (DIC) overnight at room temperature. For TNB-conjugated peptides, the N terminus was acetylated with DMF:acetic anhydride:DIEA (7:2:1, v/v/v) overnight at room temperature. Subsequently, Cys(S-tbutyl) was deprotected using 20% β-ME at room temperature overnight. Cysteine activation was done with Ellman’s reagent overnight in ethanol:DMF (3:1, v/v) solution. The final peptides were deprotected and cleaved off the solid support overnight (TNB−R_10_) or for 1 hour (FITC-LPETGG) with 95% TFA, 2.5% TIS and 2.5% water. The peptide was precipitated in diethyl ether. Purification was done using HPLC with a gradient of 10−50% acetonitrile (CH_3_CN) in water, both containing 0.1% TFA over 50 minutes. The HPLC purification yielded an A (white) or B (yellow-green) TFA salt—A: 23 mg, 0.0114 mmol, 23%; HR-MS [M + 3H]^3+^ exp.: 738.0032; calc.: 738.0696; B: 12 mg, 0.0129 mmol, 25%; HR-MS [M + H]^+^ exp.: 465.6764; calc.:465.6795. Analytical data are presented in Supplementary Fig. 10.

### NB1 expression and purification

BL21(DE3) cells were transformed with the corresponding plasmid. A streak of colonies from an LB agar plate was used to inoculate 150 ml of preculture in carbenicillin containing TB medium and incubated overnight at 37 °C under agitation. Six liters of carbenicillin containing TB medium was supplemented with 0.1% glucose and 2 mM MgCl_2_ and inoculated overnight with the preculture to a starting optial density at 600 nm (OD_600_) = 0.1. Cultures were incubated at 37 °C at 180 r.p.m. until an OD_600_ = 0.7 was reached. Cultures were then induced with 1 mM adding isopropyl β-D-1-thiogalactopyranoside (IPTG) and further incubated overnight at 28 °C and 150 r.p.m. Expression cultures were harvested by centrifugation (5,000*g*, 10 minutes, 4 °C) and resuspended in cold lysis buffer (0.2 M Tris (pH 8.0), 0.5 M sucrose and 1 mM PMSF) corresponding to pellet weight (1:1, v/w). Solution was homogenized and stirred for 1 hour at 4 °C. Double the original amount of four-times-diluted lysis buffer was added and further stirred for 45 minutes at 4 °C. To remove cell debris and whole cells and clear the lysate, cells were centrifuged (debris centrifugation; 25,000*g*, 4 °C, 30 minutes). The supernatant was reduced for 30 minutes with 1−2 mM dithiothreitol (DTT).

Then, 1.5 ml of Indigo beads was equilibrated with 50 mM sodium phosphate buffer (pH 7.0) containing 1 M NaCl and 10 mM imidazole. The clear lysate was incubated with Indigo beads (PureCube 100 INDIGO Ni-Agarose; Cube Biotech) for 1 hour at room temperature. The beads were collected in a plastic column and washed with 50 mM sodium phosphate buffer (pH 7.0) containing 1 M NaCl, 10 mM imidazole and 50 mM sodium phosphate buffer (pH 6.0) containing 1 M NaCl. The nanobody was eluted with 0.05 M CH_3_COONa and 1 M NaCl (pH 4.5) into 1 M Tris-HCl (pH 7.5). A second elution was performed with 0.05 M CH_3_COONa (pH 4.5), 1 M NaCl and 500 mM imidazole into 1 M Tris-HCl (pH 7.5). The solutions were combined. TEV cleavage was performed overnight (1:10, w/w) in dialysis against 20 mM HEPES (pH 7.5), 150 mM NaCl and 10% glycerol at room temperature. The uncleaved nanobody, His-Tag and TEV were removed after reduction with 1−2 mM DTT using Indigo beads using storage buffer (20 mM HEPES (pH 7.5), 150 mM NaCl and 10% glycerol). The nanobody was concentrated and stored at −80 °C. Purity was assessed by gel electrophoresis and HR-MS (Supplementary Fig. 2)

The nanobdoy sequence based on a previously described sequence^32^ was as follows: QVQLQESGGGLVQAGGSLRLSCAASGSIFRIDAMGWYRQAPGKQRELVAHSTSGGSTDYADSVKGRFTISRDNAKNTVYLQMNSLKPEDTAVYYCNADVRTRWYASNNYWGQGTQVTVSSGSGSC.

### GBP1 expression and purification

The GBP1 plasmid was a gift from Heinrich Leonhard^51^. GBP1 was expressed and purified as a DnaE intein and a chitin-binding domain (pTXB1 vector system) fusion protein similarly to a previously published protocol^17^. In brief, the corresponding plasmid was transformed into T7 express cells (New England Biolabs) and grown overnight at 37 °C in 5 ml of LB medium. One milliliter of this preculture was used to inoculate 250 ml of LB medium culture containing ampicillin. The culture was incubated at 37 °C to an OD_600_ = 0.6. Protein expression was induced with 1 mM IPTG, and the culture was further incubated for 16 hours at 18 °C. Cells were harvested by centrifugation (4,000*g*, 15 minutes, 4 °C). Cells were resuspended in lysis buffer (20 mM Tris-HCl (pH 8.5), 500 mM NaCl, 1 mM EDTA, 0.1% Triton X-100, 100 µg ml^−1^ lysozyme and 25 µg ml^−1^ DNAse I) and lysed by sonication (3 × 2 minutes, 30% amplitude), followed by debris centrifugation (25,000*g*, 30 minutes, 4 °C). The supernatant was loaded on chitin agarose beads, equilibrated in EPL buffer (20 mM Tris-HCl (pH 8.5) and 500 mM NaCl) and washed with 20 columns of EPL buffer. The intein cleavage was performed in the presence of cysteine to obtain GBP1 with a C-terminal cysteine residue. Therefore, the reaction was performed on the column with 1 mM cysteine in 20 mM Tris-HCl (pH 8.5), 500 mM NaCl and 100 mM sodium 2-mercaptoethanesulfonate for 16 hours while shaking at room temperature. The protein was subsequently washed off the column using EPL buffer. Further purification of the reaction mixture was performed by size exclusion chromatography using a Bio-Rad FPLC system on a Superdex 75 16/60 column in 5 mM HEPES (pH 7.5), 140 mM NaCl, 2.5 mM KCl and 5 mM glycine. Protein aliquots were shock frozen and stored at −70 °C.

The sequence of GBP1−intein was as follows (GBP1 sequence underlined): MADVQLVESGGALVQPGGSLRLSCAASGFPVNRYSMRWYRQAPGKEREWVAGMSSAGDRSSYEDSVKGRFTISRDDARNTVYLQMNSLKPEDTAVYYCNVNVGFEYWGQGTQVTVSSAAACITGDALVALPEGESVRIADIVPGARPNSDNAIDLKVLDRHGNPVLADRLFHSGEHPVYTVRTVEGLRVTGTANHPLLCLVDVAGVPTLLWKLIDEIKPGDYAVIQRSAFSVDCAGFARGKPEFAPTTYTVGVPGLVRFLEAHHRDPDAQAIADELTDGRFYYAKVASVTDAGVQPVYSLRVDTADHAFITNGFVSHATGLTGLNSGLTTNPGVSAWQVNTAYTAGQLVTYNGKTYKCLQPHTSLAGWEPSNVPALWQLQ*.

### Protein conjugation

For fluorescent N-terminal labeling of NB1, sortase A reaction was performed. Therefore, a reaction was set up with 35 nmol of purified NB1, 1.1 eq. of sortase A 5 M and 20 eq. of FITC-LPETGG peptide in sortase buffer (50 mM Tris-HCl (pH 7.5), 150 mM NaCl, 0.01 mM CaCl_2_ and 10% glycerol). The reaction was performed at room temperature for 20 minutes under mild agitation. His-tagged sortase A 5 M was removed via Ni-NTA. Excess peptide was removed by desalting in spin column using a ZebaSpin 7-kDa molecular-weight-cutoff (MWCO) spin column (Thermo Fisher Scientific) against protein buffer. Sixteen nmol of nanobody was obtained after labeling and desalting.

For the CPP conjugation, 100 nmol of cysteine-containing nanobodies was reduced for 20 minutes with 1−2 µmol of DTT. DTT was removed by desalting in spin column using a ZebaSpin 7-kDa MWCO spin column against protein buffer. Then, 3−5 eq. of TNB−R_10_ was added directly after desalting. The solution was incubated overnight at 4 °C under gentle agitation. Removal of excess TNB−R_10_ was done by desalting in spin column using a ZebaSpin 7-kDa MWCO spin column against protein buffer. Fifty-six nmol of nanobody could be obtained after conjugation purification. Conjugation success was determined by gel electrophoresis and HR-MS (Supplementary Fig. 2).

### Mammalian cell culture

All cell lines were grown at 37 °C and 5% CO_2_ in a humidified atmosphere. Cells were split at 70−90% confluency and used in passages 4−15. HeLa cells were obtained from the American Type Culture Collection (cat. no. CCL-2). HEK cells were obtained from the German Collection of Microorganisms and Cell Cultures (DSMZ) (cat. no. ACC 635). HeLa and HEK cells were grown in DMEM 4.5 g l^−1^ glucose + 10% FCS. A549 cells were obtained from the DSMZ (cat. no. ACC 107). A549 cells were grown in DMEM/Ham’s F-12 + 10% FCS. F508del-CFTR-overexpressing CFBE41o- cells were generously provided by Eric J. Sorscher (University of Alabama)^67^. Cells were cultured in minimum essential medium enriched with 10% FBS, 10 mg ml^−1^ glutamine, 100 U ml^−1^ penicillin, 100 µM ml^−1^ streptomycin and 4 µg ml^−1^ puromycin in a humidified incubator at 37 °C with 5% CO_2_. For all cell lines, no additional authentication was performed. Cell profiles were validated by the manufacturers.

### Cellular uptake experiments

For microscopy experiments, 50,000 cells per well were seeded in an eight-well ibidi glass-bottom plate. Cells were incubated for 24 hours at 37 °C with 5% CO_2_ to settle. Cells were washed once with FluoroBrite DMEM without FCS or glutamine before addition of protein samples (FITC−NB1−R_10_) and CPP additive (TNB−R_10_) in FluoroBrite DMEM without FCS or glutamine. Cells were incubated for 1 hour at 37 °C and 5% CO_2_. The cells were then washed three times with FluoroBrite DMEM with 10% FCS. For 1-hour uptake experiments, cells were counterstained with 10 µg ml^−1^ Hoechst 33342 in FluoroBrite DMEM with 10% FCS for 10 minutes and imaged in FluoroBrite DMEM with 10% FCS. For 24-hour uptake experiments, cells were further incubated for 23 hours in growth medium prior to counterstaining with Hoechst 33342 as described previously and imaged in FluoroBrite DMEM with 10% FCS. Imaging was done using a Zeiss LSM 780 confocal microscope with a ×63, 1.4 numerical aperture Plan-Apochromat lens at room temperature. 5 × 5 tile scans were acquired alongside single tile images. Image analysis and processing were performed with Fiji software^68^.

For 4 °C microscopy experiments, 50,000 cells per well were seeded in an eight-well ibidi glass-bottom plate. Cells were incubated for 24 houra at 37 °C with 5% CO_2_ to settle. Prior to incubation, cells were cooled down to 4 °C for 30 minutes. Cells were washed once with cold FluoroBrite DMEM without FCS or glutamine before addition of cold protein samples (FITC−NB1−R_10_) and CPP additive (TNB−R_10_) in FluoroBrite DMEM without FCS or glutamine. Cells were incubated for 1 hour at 4 °C. The cells were then washed three times with cold 0.5 mg ml^−1^ heparin in PBS. Cells were counterstained with cold 10 µg ml^−1^ Hoechst 33342 in FluoroBrite DMEM with 10% FCS for 10 minutes at 4 °C and imaged in FluoroBrite DMEM with 10% FCS at room temperature. Imaging was done using a Zeiss LSM 710 confocal microscope with a ×63, 1.4 numerical aperture Plan-Apochromat lens at room temperature. Image analysis and processing were performed with Fiji software^68^.

For endosomal co-localization experiments, 50,000 cells per well were seeded in an eight-well ibidi glass-bottom plate. Cells were incubated for 24 hours at 37 °C with 5% CO_2_ to settle. Cells were preincubated for 30 minutes with 1 nM SiR-lysosome (Spirochrome, SC012). Cells were washed once with FluoroBrite DMEM without FCS or glutamine before addition of protein samples (FITC−NB1−R_10_) and CPP additive (TNB−R_10_) in FluoroBrite DMEM without FCS or glutamine. Cells were incubated for 1 hour at 37 °C and 5% CO_2_. The cells were then washed three times with FluoroBrite DMEM with 10% FCS. For 1-hour uptake experiments, cells were counterstained with 10 µg ml^−1^ Hoechst 33342 in FluoroBrite DMEM with 10% FCS for 10 minutes and imaged in FluoroBrite DMEM with 10% FCS. For 24-hour uptake experiments, cells were further incubated for 23 hours in growth medium prior to counterstaining with Hoechst 33342 as described before and imaged in FluoroBrite DMEM with 10% FCS. Imaging was done using a Zeiss LSM 710 confocal microscope with a ×63, 1.4 numerical aperture Plan-Apochromat lens at room temperature. 5 × 5 tile scans were acquired alongside single tile images.

Image analysis and processing were performed with Fiji software^68^. Representative images were selected. Images were cropped, and the minimum and maximum displayed intensity values were adjusted to increase contrast. When images were compared, the contrast adjustments were the same for all images. Scale bars were added.

### Co-localization experiments

Inducible CFBE41o- mCherry−Flag−F508del-CFTR cells were a kind gift from Margarida D. Amaral^49,69,70^. The cells were cultured in DMEM high glucose supplemented with 10% FCS, 10 μg ml^−1^ blasticidin and 2 μg ml^−1^ puromycin. For co-localization experiments, 50,000 cells per well were seeded in an eight-well ibidi glass-bottom plate and left to settle overnight (37 °C and 5% CO_2_). mCherry−Flag−F508del-CFTR expression was induced with 1 µg ml^−1^ doxycycline DMEM high glucose supplemented with 10% FCS for 24 hours. Cells were washed once with FluoroBrite DMEM without FCS or glutamine before addition of 10 µM FITC−NB1−R_10_ or FITC−GBP1−R_10_ in the presence of 10 µM TNB−R_10_ in FluoroBrite DMEM without FCS or glutamine. Cells were incubated for 1 hour at 37 °C and 5% CO_2_. The cells were then washed three times with FluoroBrite DMEM with 10% FCS and incubated in DMEM high glucose supplemented with 10% FCS and 1 µg ml^−1^ doxycycline for 16 hours. Cells were counterstained with 10 µg ml^−1^ Hoechst 33342 in FluoroBrite DMEM with 10% FCS for 10 minutes and imaged in FluoroBrite DMEM with 10% FCS. Imaging was done using a Zeiss LSM 780 confocal microscope with a ×63, 1.4 numerical aperture Plan-Apochromat lens at room temperature. 5 × 5 tile scans were acquired alongside single tile images.

Image analysis and processing were performed with Fiji software^68^. Representative images were selected. Images were cropped, and the minimum and maximum displayed intensity values were adjusted to increase contrast. When images were compared, the contrast adjustments were the same for all images. Scale bars were added. For Pearson’s correlation coefficient analysis, 10−15 single cells per treatment condition from at least three different regions of a well were manually selected. For selection, cells that showed NB1 uptake as well as induced mCherry signal were chosen. Cells that were overexposed were excluded. Pearson’s correlation coefficient was calculated for each cell using the BIOP JACoP Plugin in Fiji using an automatic threshold (mean). Pearson’s correlation coefficient was calculated as *r* ± s.d.

### Nanobody cell content assessment

#### Total cell content

In total, 150,000 CFBE41o- cells per well were seeded into a 12-well cell culture dish and incubated for 24 hours (25-hour samples) or 48 hours (1-hour samples) at 37 °C and 5% CO_2_. Cells were washed once with FluoroBrite DMEM without FCS or glutamine before addition of protein samples (FITC−NB1−R_10_) and CPP additive (TNB−R_10_) in FluoroBrite DMEM without FCS or glutamine. Cells were incubated for 1 hour at 37 °C and 5% CO_2_. The cells were then washed with 0.5 mg ml^−1^ heparin in PBS and either further incubated for 24 hours in growth medium (25-hour samples) or directly further used. Cells were washed once with PBS and harvested with Accutase and pelleted at 330*g*, 5 minutes. Cells were washed with PBS, and cell count was measured. Cells were repelleted and treated with 50 µl of 2× Laemmli buffer and boiled for 10 minutes at 95 °C. Ten microliters of sample was analyzed via gel electrophoresis together with defined concentration of FITC−NB1. Gel images were taken on a ChemiDoc MP system (Bio-Rad) using Image Lab software (version 5.1, Bio-Rad). Band fluorescence intensity was analyzed using Fiji. Measured gel intensities of the defined concentration nanobody samples were used to create a standard curve. This curve was used to measure the amount of nanobody in the lysate samples. The results were then normalized to the measured cell number of the sample and stated in nanograms per 1,000 cells.

#### Cytosolic cell content

Cytosolic content was determined adapting a previously published protocol by Wissner et al.^44^. In total, 400,000 CFBE41o- cells per well were seeded into a six-well cell culture dish and incubated for 24 hours (25-hour samples) or 48 hours (1-hour samples) at 37 °C and 5% CO_2_. Cells were washed once with FluoroBrite DMEM without FCS or glutamine before addition of protein samples (FITC−NB1−R_10_) and CPP additive (TNB−R_10_) in FluoroBrite DMEM without FCS or glutamine. Cells were incubated for 1 hour at 37 °C and 5% CO_2_. The cells were then washed with 0.5 mg ml^−1^ heparin in PBS and either further incubated for 24 hours in growth medium (25-hour samples) or directly further used. Cells were washed once with PBS, harvested with Accutase and pelleted at 330*g*, 5 minutes. Cells were washed with PBS, and cell count was measured. Cells were repelleted and washed one more time with PBS followed by a wash with precooled isotonic sucrose (290 mM sucrose, 10 mM imidazole (pH 7.0), 1 mM DTT and one cOmplete protease inhibitor cocktail tablet per 10 ml). Cells were resuspended in 100 µl of isotonic sucrose and transferred to 1.4-mm ceramic beads containing 0.5-ml tubes (Omni International). Cells were homogenized by bead ruptor using a disruptor genie (Scientific Industries) at speed 15,000 r.p.m. for 8 seconds. A centrifugation at 350,000*g* for 30 minutes at 4 °C was performed to separate cytosolic content and organelles. Cytosolic fractions were boiled with 4× Laemmli buffer at 95 °C for 5 minutes and separated by SDS-PAGE. Gel images were taken on a ChemiDoc MP system (Bio-Rad) using Image Lab software (version 5.1, Bio-Rad). Band fluorescence intensity was analyzed using Fiji. Measured gel intensities of the defined concentration nanobody samples were used to create a standard curve. This curve was used to measure the amount of nanobody in the lysate samples. The results were then normalized to the measured cell number of the sample and stated in nanograms per 1,000 cells.

### Endosome rupture assay

In total, 3,000,000 CFBE41o- cells were seeded into a 60-mm cell culture dish and incubated for 24 hours at 37 °C and 5% CO_2_. The next day, cells were transfected using 10 µg of pEGFP-hGal3, a gift from Tamotsu Yoshimori (Addgene plasmid no. 73080; http://n2t.net/addgene:73080; RRID: Addgene_73080)^48^ using Lipofectamin 3000 and P3000 Reagent following the manufacturerʼs protocol for transient transfection. The next day, transfected cells were harvested and seeded into eight-well microscopy (50,000 cells per well) slides. On the next day, cells were treated with 10−75 µM FITC−NB1−R_10_ in the presence of 10−30 µM CPP additive or LLOMe (1 mM) as positive control for 30 minutes. Cells were washed twice and imaged in FluoroBrite DMEM supplemented with 10% FCS and 2 mM glutamine. Imaging was done using a Zeiss LSM 710 confocal microscope with a ×63, 1.4 numerical aperture Plan-Apochromat lens at room temperature. Image analysis and processing were performed with Fiji software^68^.

### Cell viability and membrane integrity assay

For cell viability measurements using mitochondrial dehydrogenase (WST-1) assay and membrane integrity assay using LDH release assay, 15,000 cells per well were seeded in a 96-well plate and incubated for 24 hours at 37 °C and 5% CO_2_ to settle. Cells were washed once with FluoroBrite DMEM without FCS or glutamine. Cells were treated with NB1−R_10_ or FITC−NB1−R_10_ in indicated concentrations together with 10−30 µM TNB−R_10_ in FluoroBrite DMEM without FCS or glutamine for 1 hour at 37 °C and 5% CO_2_. For the LDH assay, 50 µM supernatant was transferred into a black 96-well plate. Then, 50 µl of CytoTox-ONE Homogeneous Membrane Integrity Assay reagent (Promega) was added and incubated for 10 minutes at room temperature. Fluorescence intensity (excitation/emission: 560/590 nm) was measured using an M200Pro plate reader (TECAN). For WST-1 assay, cells were washed with growth medium after incubation and either submitted to WST-1 measurements or incubated for 24 hours at 37 °C and 5% CO_2_ further. Then, 10 µl of WST-1 reagent was added to each well and incubated for 90 minutes at 37 °C and 5% CO_2_. Absorbance at 460 nm was measured using an M200Pro plate reader (TECAN).

### Culture and treatment of CF bronchial epithelial cells

Prior to seeding, CFBE41o- cells underwent trypsinization and were passaged at a ratio of 1:2 while puromycin was removed from the culture medium. The next day, cells were trypsinized, and 500,000 cells were seeded onto Snapwell inserts (Corning, 3407). The CFBE41o- monolayers were maintained at a liquid−liquid interface. Upon reaching transepithelial electrical resistance (TEER) values of ≥600 Ω × cm^2^, the cells were subjected to apical treatment with either minimum essential medium alone (vehicle control) or different concentrations of NB1−R_10_ and TNB−R_10_ additive for 24 hours. In a subset of experiments, cells were additionally treated with 0.076% DMSO as vehicle control or elexacaftor (3 µM) and tezacaftor (18 µM) in the basolateral medium for 24 hours. For temperature correction of F508del-CFTR, cultures were placed in a humidified incubator at 27 °C with 5% CO_2_ for 24 hours.

### Study approval and study population

The study was approved by the ethics committee of the Charité − Universitätsmedizin Berlin (EA2/161/20). Written informed consent was obtained from all participants.

### Culture and treatment of primary airway epithelial cells from healthy individuals and patients with CF

Primary airway epithelial cells were derived from nasal brushings of three healthy individuals and three patients with CF homozygous for the *F508del* allele, using an established differentiation protocol^61^. After expansion, cells at passage 2 were seeded onto human placental type IV collagen-coated Snapwell or Transwell inserts (Corning, 3407 or 3460) at a density of 200,000 cells per cm^2^. Epithelial cultures were differentiated at the air–liquid interface for 3−4 weeks in PneumaCult-ALI medium (STEMCELL Technologies). Fully differentiated cultures were then apically treated with either NB1−R_10_/TNB−R_10_ diluted in PBS or a vehicle control (PBS alone) for 3 hours, followed by aspiration of the apical treatment medium. In a subset of experiments, cells were treated with DMSO as vehicle control or elexacaftor and tezacaftor as described above.

### Ussing chamber measurements

*I*_sc_ measurements in CFBE41o- and primary nasal epithelial cultures were conducted using EasyMount Ussing chambers (Physiologic Instruments) with a chloride gradient (145 mM basolateral and 5 mM apical) using a voltage-clamp setup as previously described^71^. In brief, culture inserts were first equilibrated for 15−20 minutes in the presence of amiloride (100 µM). To assess CFTR-mediated chloride secretion, forskolin (10 µM) and 3-isobutyl-1-methylxanthin (IBMX, 100 µM) were administered both apically and basolaterally, followed by CFTRinh-172 (20 µM) apically. In a subset of experiments, ivacaftor (5 µM) was administered after forskolin/IBMX to assess potentiation of CFTR-mediated currents.

### Immunoblot analysis

Immunoblot analysis was performed on CFBE41o- whole-cell lysates as previously described^61^. In brief, cells were scraped directly into 80 µl of RIPA buffer (Thermo Fisher Scientific) containing 2% sodium dodecyl sulfate and 1 mM PMSF protease inhibitor (Thermo Fisher Scientific). For western blotting, 5 µl of lysate supernatants was mixed with Laemmli buffer and 0.05% β-ME at 37 °C for 30 minutes and then subjected to electrophoresis on 4−20% gradient polyacrylamide gels (Bio-Rad). Proteins were transferred to PVDF membrane and processed for western blotting by using 1:500 dilution of CFTR antibody 596 (provided by John Riordan, University of North Carolina at Chapel Hill, via the CF Foundation Antibody Distribution Program, lot: 596TJ10028520240213) and 1:200 dilution of β-actin antibody (Sigma-Aldrich, A1978, lot: 0000086303). The secondary antibody used for CFTR and β-actin antibody was a 1:5,000 dilution of HRP-conjugated goat polyclonal anti-mouse antibody (Dako Denmark, P0047, lot: 41236467). The HRP signal was detected using Pierce ECL Western Blotting Substrate (Thermo Fisher Scientific) and imaged using the ChemiDoc MP system (Bio-Rad). Fiji software was used for densitometric analysis^68^. To assess CFTR maturation, the relative amount of CFTR C-band protein was normalized to loading control (β-actin).

### Flow cytometry

For the flow cytometry experiments, CFBE41o- cells were seeded with 150,000 cells in a 24-well plate. Cells were incubated for 24 hours at 37 °C and 5% CO_2_ to settle and then treated with 75 µM FITC−NB−R_10_ and 30 µM TNB−R_10_ or growth medium for 1 hour in FluoroBrite DMEM without FCS and washed after 1 hour. They were further incubated for 16 hours in growth medium. For the temperature correction control, cells were incubated in growth medium at 27 °C and 5% CO_2_ for 17 hours. Multiple wells per experiment were treated under the same conditions. Cells were detached with Accutase, and samples were prepared at 4 °C. Therefore, cells were washed twice with cold blocking buffer (PBS containing 1% BSA). Cells were stained with primary antibody against an extracellular loop peptide sequence of CFTR (CFTR Monoclonal Antibody (CF3), Invitrogen, MA1-935, lot: XL366836, 1:200), isotype control (Mouse IgM Isotype Control (11E10), eBioscience, Invitrogen, 14-4752-82, 1:200) or medium as untreated control, for 30 minutes on ice. Cells were washed twice with blocking buffer and stained with secondary antibody (goat anti-mouse IgM (heavy chain) secondary antibody, Alexa Fluor 647, Invitrogen, A21238, lot: 2566350, 10 µg ml^−1^) or medium for untreated control for 30 minutes on ice. Cells were washed twice with blocking buffer and once with PBS. Cells were resuspended in PBS and measured on an LSRFortessa (BD Biosciences) flow cytometer using LSRFortessa Cell Analyzer software with 10,000 events per measurement. FITC and Alexa Fluor 647 fluorescence were measured alongside forward scatter (FWS) and side scatter (SWS). Cell fragments and multiplets were removed in the analysis by gating using FlowJo software. Subsequently, the FITC channel in a medium-treated sample was used to determine the FITC-negative population. Higher fluorescence values in FITC were deemed as the FITC-positive population. The gating strategy is illustrated in Supplementary Fig. 6b for a nanobody and medium-treated sample. For normalization of Alexa Fluor 647 fluorescence values, mean fluorescence value of a given sample was divided by the mean fluorescence value of the isotype control of medium-treated cells.

### Confocal fluorescence live-cell microscopy for cell surface CFTR staining

In total, 15,000 cells per well were seeded in a 96-well plate (thin bottom; Corning) and incubated for 24 hours at 37 °C and 5% CO_2_ to settle and then treated with 75 µM FITC−NB−R_10_ and 30 µM TNB−R_10_ or growth medium for 1 hour in FluoroBrite DMEM without FCS and washed after 1 hour. They were further incubated for 16 hours in growth medium. For the temperature correction control, cells were incubated in growth medium at 27 °C and 5% CO_2_ for 17 hours. Cells were washed once with PBS and incubated in blocking buffer (PBS containing 1% BSA). Cells were stained with primary antibody (CFTR Monoclonal Antibody (CF3), Invitrogen, MA1-935, lot: XL366836, 1:200), isotype control (Mouse IgM Isotype Control (11E10), eBioscience, Invitrogen, 14-4752-82, 1:200) or medium as untreated control for 30 minutes on ice. Cells were washed twice with PBS and stained with secondary antibody (goat anti-mouse IgM (heavy chain) secondary antibody, Alexa Fluor 647, Invitrogen, A21238, lot: 2566350, 10 µg ml^−1^) or medium for untreated control for 30 minutes on ice. Cells were counterstained with 10 µg ml^−1^ Hoechst 33342 in FluoroBrite DMEM with 10% FCS for 10 minutes and imaged in FluoroBrite DMEM with 10% FCS. Imaging was done using a Zeiss LSM 780 confocal microscope with a ×63, 1.4 numerical aperture Plan-Apochromat lens at room temperature. Image analysis and processing was performed with Fiji software^68^. Representative images were selected. Images were cropped to present key regions; full-size images are supplied in the supplementary data. The minimum and maximum displayed intensity values were adjusted to increase contrast. When images were compared, the contrast adjustments were the same for all images. Scale bars were added.

### Confocal live-cell imaging in patient-derived airway epithelial cells

Highly differentiated patient-derived nasal epithelial cultures were treated apically with 10 µM FITC−NB1−R_10_/10 µM TNB−R_10_ in PBS for 3 hours, followed by aspiration and three times PBS wash. Twenty-four hours after treatment, live-cell nuclear staining was performed by adding 4′,6-diamidino-2-phenylindole (Hoechst, 1:15,000 dilution in PneumaCult-ALI medium) to the basolateral compartment. Confocal imaging was performed using a Leica Stellaris 8 confocal laser scanning microscope using a ×20 water immersion objective. Maximum intensity *z*-projections are shown.

### Software

Microscopy pictures were processed with ImageJ including the Fiji package. *I*_sc_ measurements were recorded using LabChart 8. Graphing and statistics were done using GraphPad Prism 10 and BioRender. Flow cytometry data were processed and analyzed using FlowJo. Figures were created with BioRender and InkScape 1.4.3.

### Statistics and reproducibility

The statistical tests and values of *n* are reported in the figure legends where appropriate. All imaging experiments were independently repeated at least once with the same results.

### Reporting summary

Further information on research design is available in the Nature Portfolio Reporting Summary linked to this article.

## Online content

Any methods, additional references, Nature Portfolio reporting summaries, source data, extended data, supplementary information, acknowledgements, peer review information; details of author contributions and competing interests; and statements of data and code availability are available at 10.1038/s41589-026-02199-w.

## Supplementary information

Supplementary Information Supplementary Figs. 1−10.

Reporting Summary

## Source data

Source Data Fig. 1 Unprocessed gel.

Source Data Fig. 3 Statistical source data.

Source Data Fig. 4 Statistical source data.

Source Data Extended Data Fig. 2 Statistical source data.

Source Data Extended Data Fig. 2 Unprocessed gels.

Source Data Extended Data Fig. 3 Unprocessed western blots.

Source Data Extended Data Fig. 4 Statistical source data.

## Data Availability

All data needed to interpret and analyze the research in the article are present in the paper and/or the Supplementary Information. All experimental data, materials and methods, analytical procedures, cell assays and copies of spectra are available in the main text and in the Supplementary Information. Raw imaging data are available upon reasonable request. Source data are provided with this paper.
