# Supplementary material for: A cell-permeable nanobody to restore F508del cystic fibrosis transmembrane conductance regulator activity
Source: Nat Chem Biol. 2026 Apr 17;22(7):1155–64. doi: 10.1038/s41589-026-02199-w (PMC13303082; doi:10.1038/s41589-026-02199-w)
Supplement: Supplementary file 1 — Supplementary Figs. 1−10. [file 41589_2026_2199_MOESM1_ESM.pdf]

nature chemical biology

Supplementary information https://doi.org/10.1038/s41589-026-02199-w

A cell-permeable nanobody to restore F508del cystic fibrosis transmembrane conductance regulator activity

In the format provided by the authors and unedited

Supplementary Information

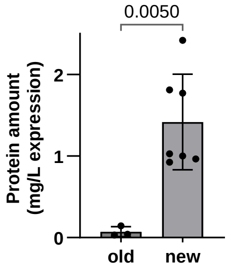

Supplementary Figure 1: Comparison of expression yields for nanobody NB1. Protein yield of nanobody per 1 L of expression was calculated as mean +/- standard deviation. A significant difference could be detected between yields obtained by the estabilished protocol and the changed protocol. The data is presented as mean of n≥3 independent samples per conditiosn represented as individual points. Statistical significance was assessed by unpaired t-test with p=0.0050

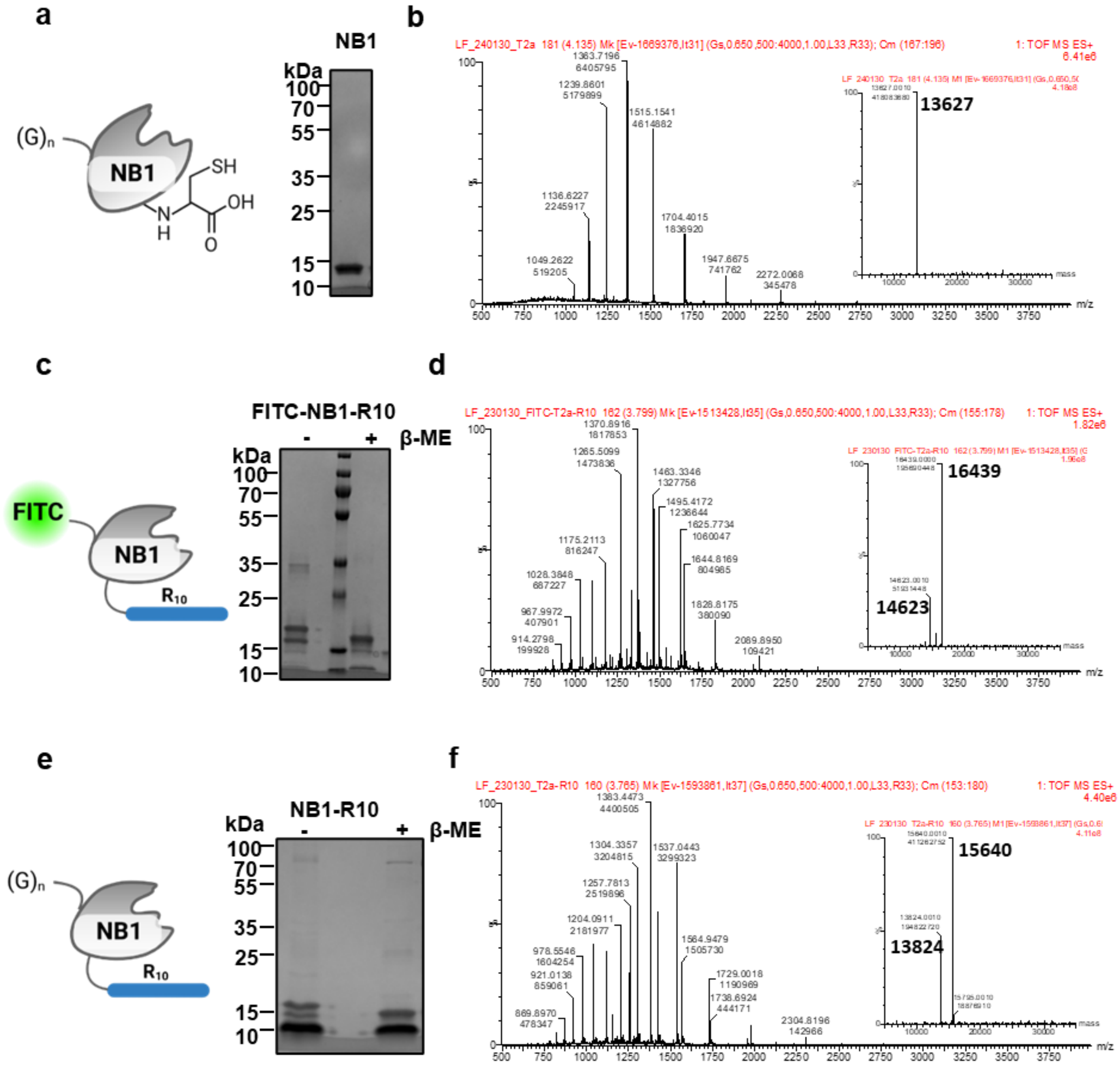

Supplementary Figure 2: Characterisation of purified nanobody conjugates. **a** SDS PAGE gel showing purified NB1 in 1x Laemmli buffer after purification. **b** HRMS spectrum of NB1 $[M+H]^+$ calc. 13627Da. **c** SDS-PAGE gel of FITC-NB1-R10 in 1x Laemmli buffer with (+) or without (-) 2.5% β-mercaptoethanol (β-ME) showing two bands without reducing conditions indicating the succesfull conjugation of CPP via disulfide. **d** HRMS spectrum of FITC-NB1-R10 $[M+H]^+$ calc. 16439 Da, FITC-NB1 $[M+H]^+$ calc. 14632 Da. **e** SDS-PAGE gel of NB1-R10 in 1x Laemmli buffer with (+) or without (-) 2.5% β-ME showing two bands without reducing conditions indicating the succesfull conjugation of CPP via disulfide. **f** HRMS spectrum of NB1-R10 $[M+H]^+$ calc. 15640 Da.

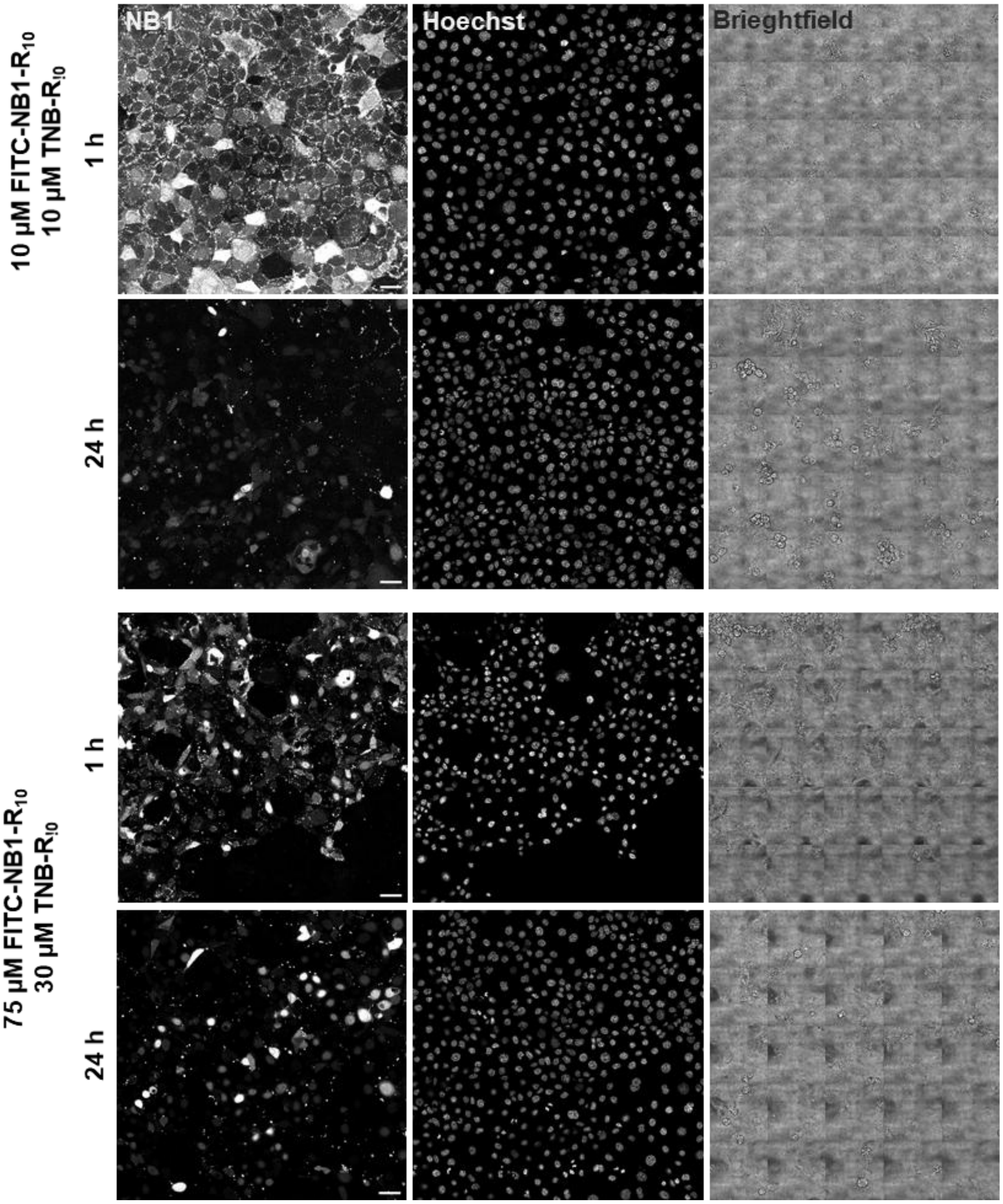

Supplementary Figure 3: Tile scan images of the ccellular delivery of a CFTR-binding nanobody. CFBE41o- cells were treated with 10 µM FITC-NB1-R_{10}/10 µM TNB-R_{10} or 75 µM FITC-NB1-R_{10}/30 µM TNB-R_{10} for 1 h in serum-free medium. Images were taken either after incubation with the nanobody for 1 h or an additional 24 h incubation in growth medium (25 h). Scale bar: 50 µm.

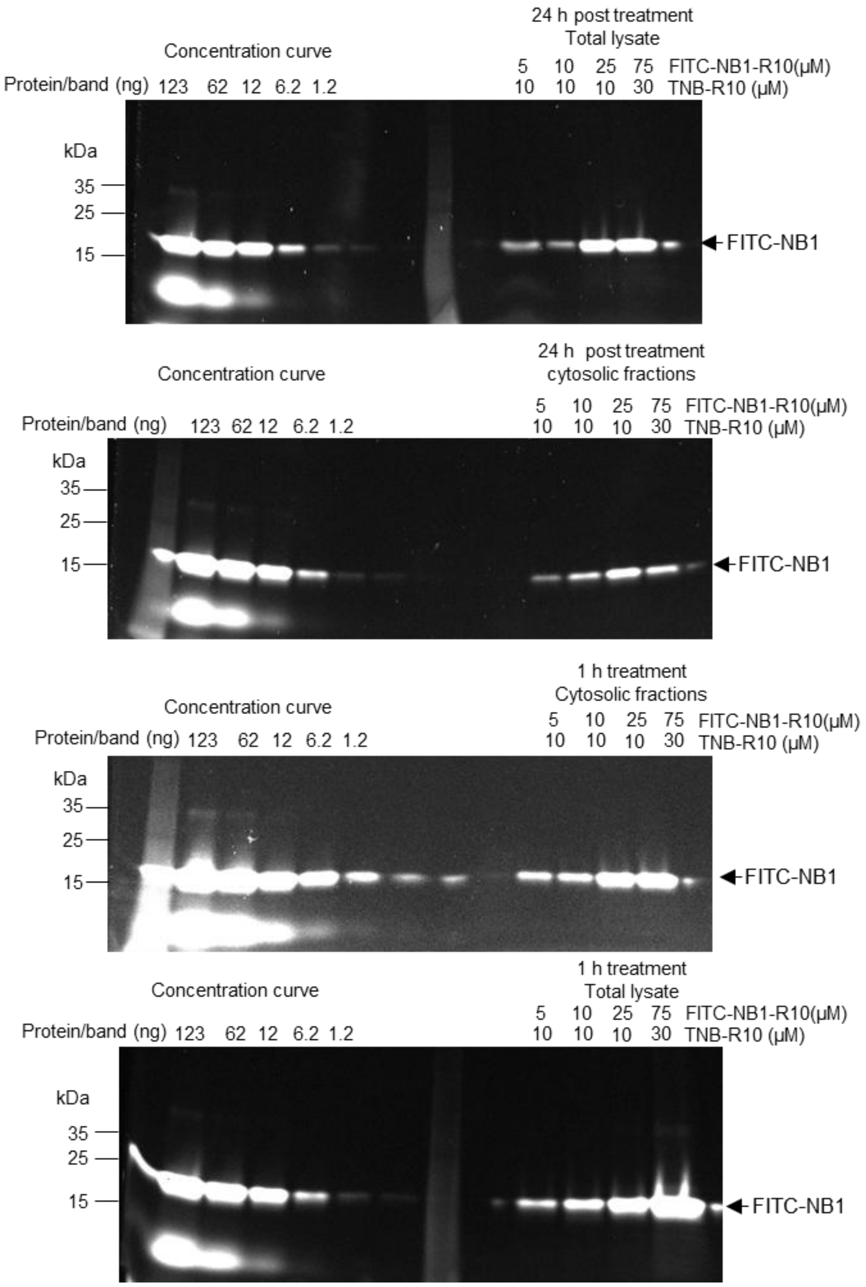

Supplemantary Figure 4: SDS-PAGE of cell treated with different concentrations of FITC-NB1-R10 and TNB-R10 as indicated. Cells were harvested and lysed after treatment (1h) or 24 h post treatement (24 h) by Laemmli buffer (total lysate) or lysed and fractioned to obtain cytosolic content (cytosolic fractions Samples with defined protein amounts of FITC-NB1-R10 per band were added on each gel to obtain a concentration curve. All samples were run under reducing conditions.

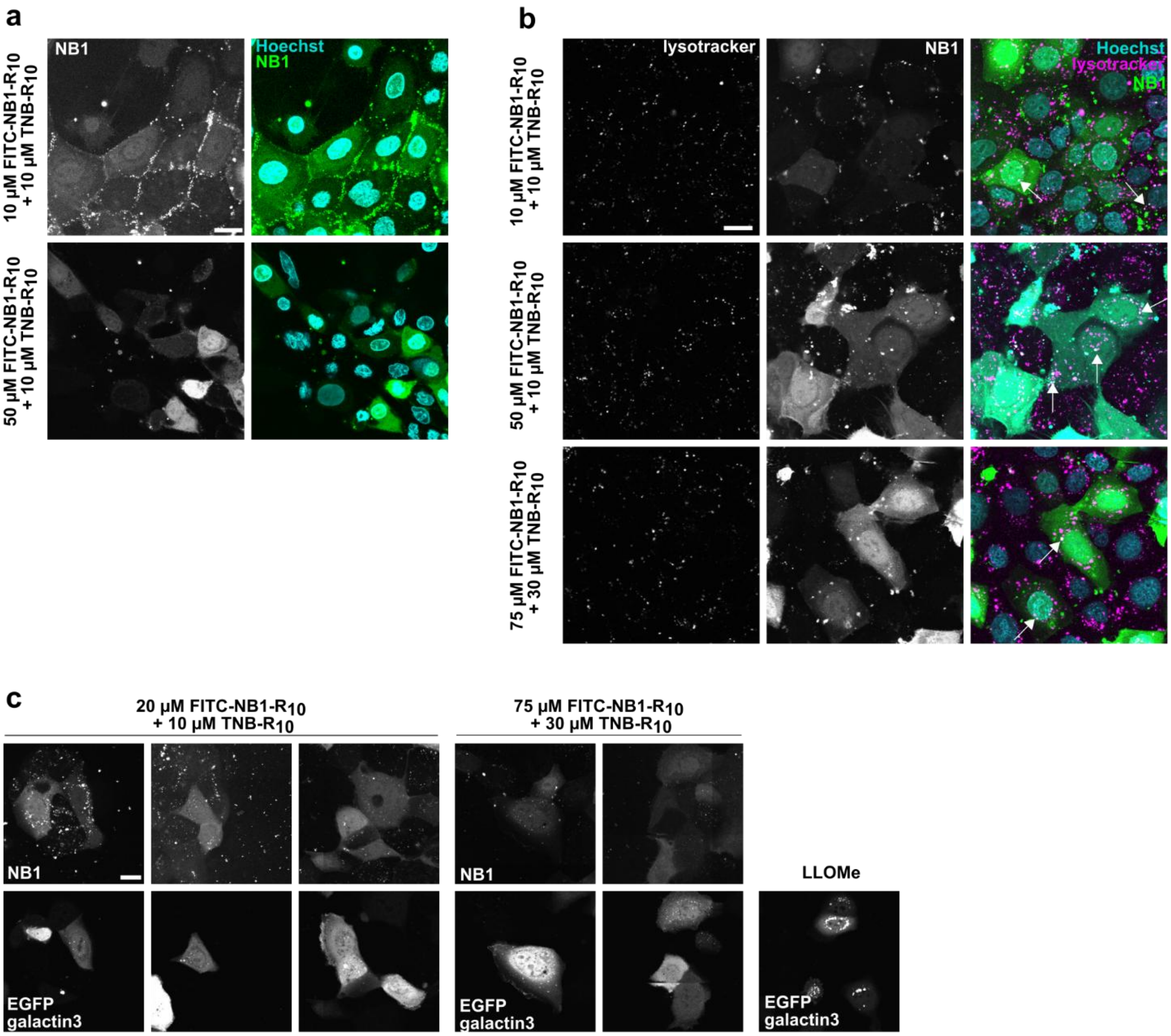

Supplementary figure 5: Investigations into cellular uptake pathways of FITC-NB1-R_{10}. **a** Cellular deliver at 4 °C. CFBE41o- cells were cooled at 4°C for 15 min and incubated with 10-50 µM FITC-NB1-R_{10}/ 10 µM TNB-R_{10} in serum-free conditions for 1 h. Cells were washed and confocal fluorescence live-cell microscopy images were taken at RT. Scale bar: 20 µM. **b** Detection of endosomal uptake. CFBE41o- cells were pre-incubated for 30 min with lysotracker and treated with 10-75 µM FITC-NB1-R_{10}/ 10-30 µM TNB-R_{10} for 1 h in serum-free medium. Confocal fluorescence live-cell microscopy images were taken. Co-localization of endosomes and nanobody containing vesicles are indicated by white arrows. Scale bar: 20 µm. **c** Endosome rupture assay. CFBE41o- cells were transfected with EGFP-galactin3 and treated with 20 µM FITC-NB1-R_{10}/ 10 µM TNB-R_{10} and 75 µM FITC-NB1-R_{10}/ 30 µM TNB-R_{10} or LLOMe as a positive control for endosomal rupture. If the uptake of NB1 results in endosome rupture, EGFP-galactin3 will aggregate at sites of ruptured endosome yielding a punctated signal (LLOMe). Scale bar: 20 µM.

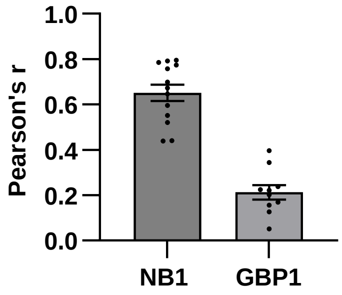

Supplementary Figure 6: Pearson’s correlation coefficient indicating co-localisation between Flag F598delCFTR-mCherry and NB1. No correclation is indicated between Flag F598delCFTR-mCherry and an unspecific control nanobody GBP1. Bar graphs represent the mean ± SEM of n≥10 cells per treatment group. Single values are represented by dots.

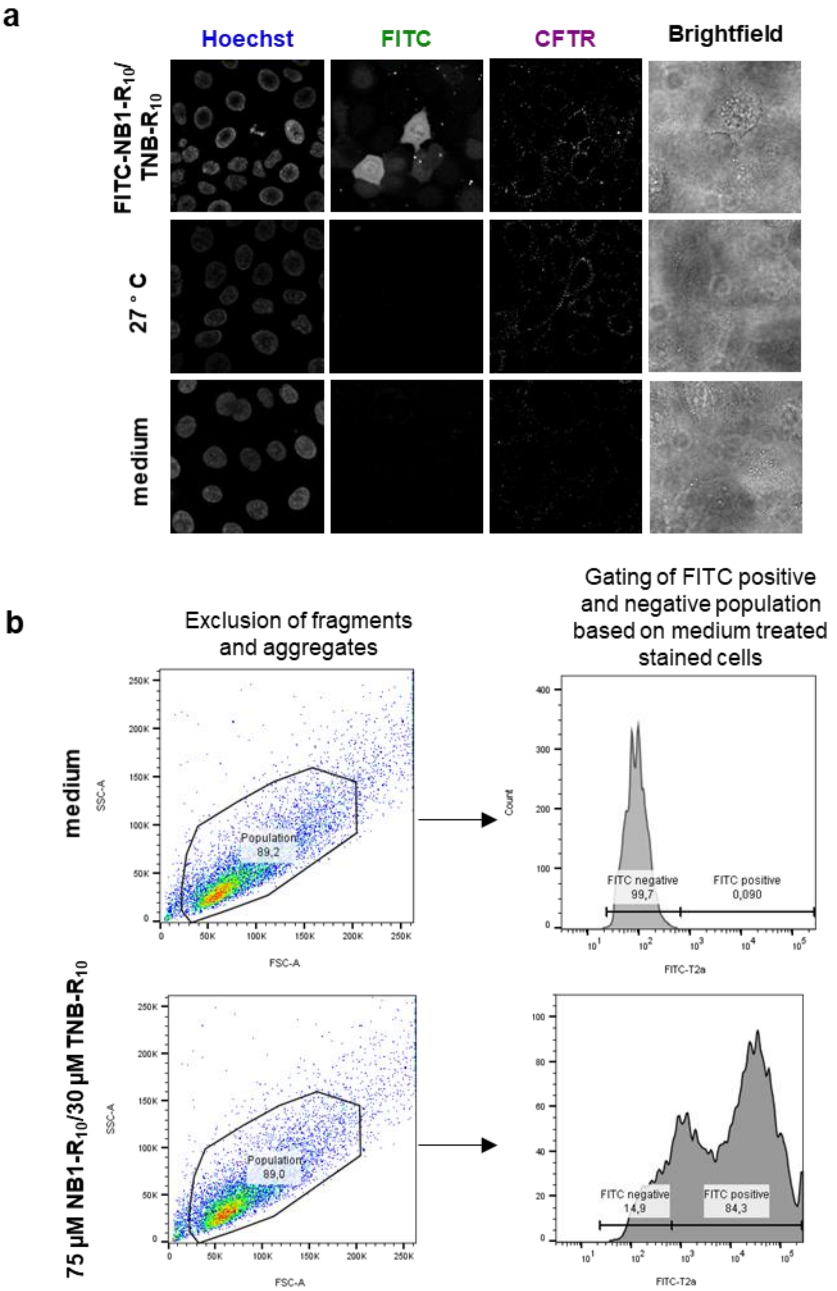

Supplementary Figure 7: Investigation of cell surface CFTR expression. **a** Live-cell confocal microscopy images of cell surface CFTR staining. CFBE41o- cells were treated for 1 h with 75 µM NB-R_{10} and 30 µM TNB-R_{10} in serum-free FluoroBrite DMEM (top row), serum-free FluoroBrite DMEM (medium) (bottom row) and subsequently incubated for 16 h in growth medium or incubated in growth medium for 16 h at 27°C as temperature control (27°C) (middle row). **b** Gating example for semi-quantitative analysis of CFTR on the cell surface by flow cytometry shown for a stained untreated sample (medium). To select the viable population a sample sideward scatter (SSC-A) and forward scatter (FSC-A) were used. The gate was set to exclude fragments and aggregates. For the nanobody treated sample only the nanobody containing (FITC positive population) was considered to determine the mean AF647 fluorescence representing the stained cell surface CFTR content. The gate for FITC negative population was set using the untreated (medium) sample. The population with higher fluorescence than the FITC negative population was gated as FITC positive population as indicated. Application of gates to a representative measurement of a stained nanobody treated sample (75 µM NB1-R_{10} + 30 µM TNB-R_{10}).

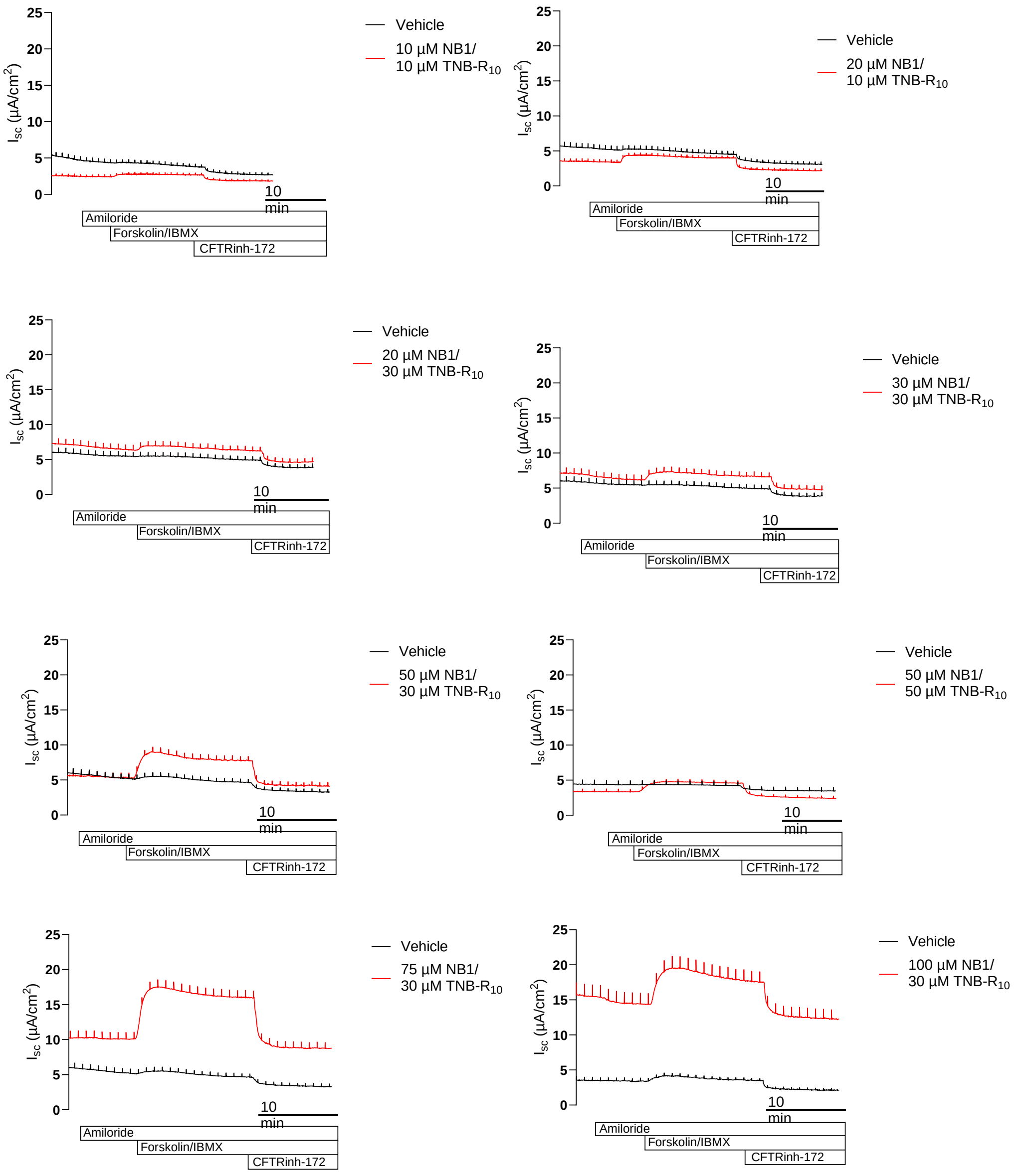

Supplemetary Fig. 8: Representative Ussing chamber tracings of transepithelial current (I_{sc}) from dose-response studies assessing the effect of NB1-R_{10}/TNB-R_{10} on functional rescue of F508del-CFTR in CFBE41o- cells.

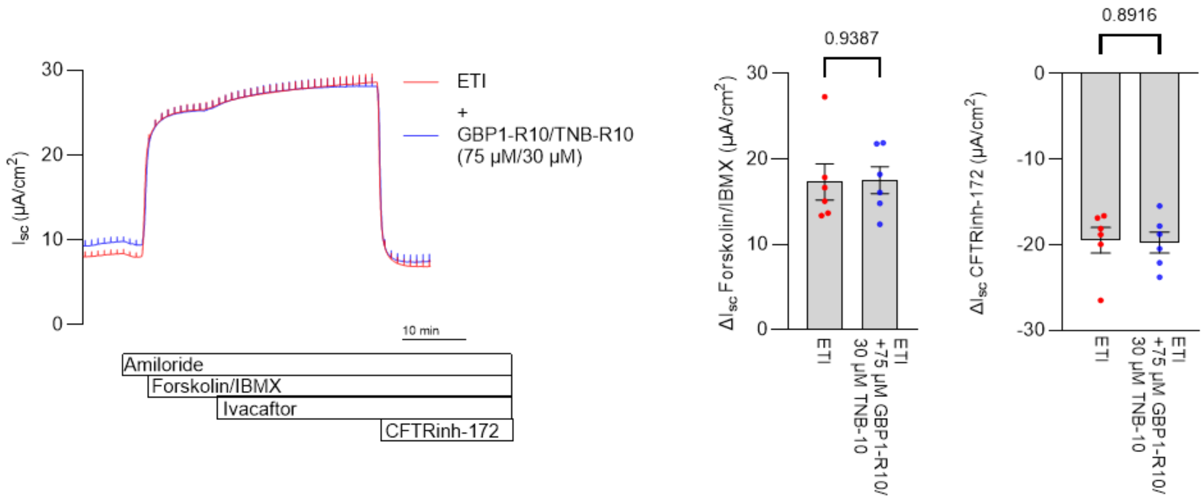

Supplementary Figure 9: Representative Ussing chamber tracing and summary of effects of ETI in the absence and presence of 75 µM GBP1 GBP1-R_{10}/30 µM TNB-R_{10} on transepithelial current (I_{sc}) in F508del-CFTR expressing CFBE41o- cells. Representative original recordings of Isc measurements. Quantification of forskolin/IBMX-induced Isc and CFTRinh-172-sensitive Isc. Bar graphs represent mean ± SEM of n≥4 samples per treatment group. Statistical significance was determined by comparing treatment groups with one-way ANOVA, p values are stated in the figure.

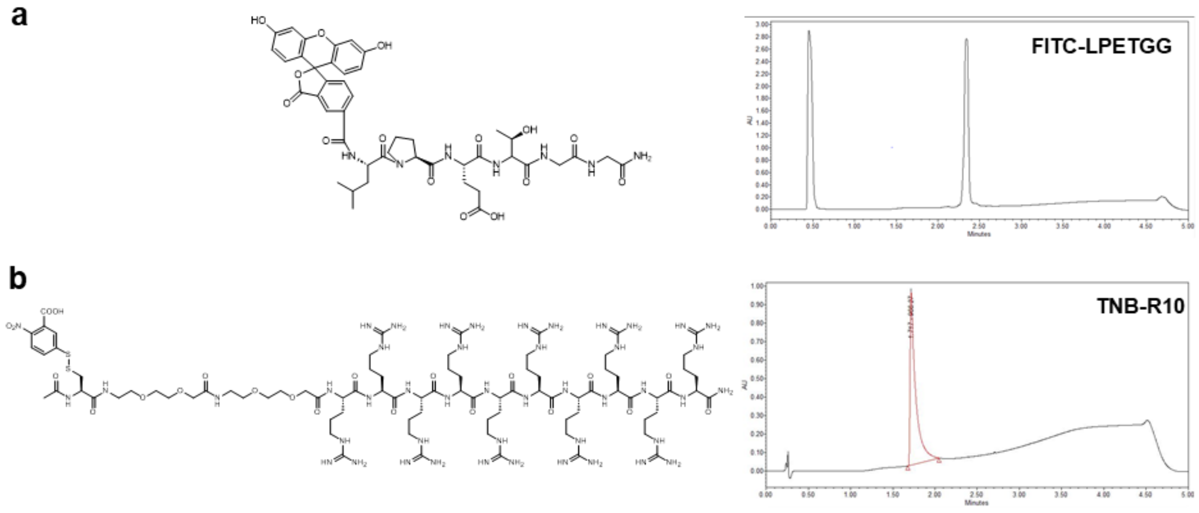

Supplementary Figure 10: Analytical data for synthetic peptides. **a** FITC-LPETGG HRMS [M+3H]3+ exp. 738.0032; calc.: 738.0696 **b** TNB-R10 HR-MS [M+H]+ exp.: 465.6764; calc.:465.6795
