# Supplementary material for: A cell-permeable nanobody to restore F508del cystic fibrosis transmembrane conductance regulator activity
Source: Nat Chem Biol. 2026 Apr 17;22(7):1155–64. doi: 10.1038/s41589-026-02199-w (PMC13303082; doi:10.1038/s41589-026-02199-w)
Supplement: Supplementary file 2 — Reporting Summary [file 41589_2026_2199_MOESM2_ESM.pdf]

nature portfolio

Corresponding author(s): Christian P.R. Hackenberger

Last updated by author(s): Feb 16, 2026

Reporting Summary

Nature Portfolio wishes to improve the reproducibility of the work that we publish. This form provides structure for consistency and transparency in reporting. For further information on Nature Portfolio policies, see our Editorial Policies and the Editorial Policy Checklist.

Statistics

For all statistical analyses, confirm that the following items are present in the figure legend, table legend, main text, or Methods section.

n/a	Confirmed	
☐	☒	The exact sample size (*n*) for each experimental group/condition, given as a discrete number and unit of measurement
☐	☒	A statement on whether measurements were taken from distinct samples or whether the same sample was measured repeatedly
☐	☒	The statistical test(s) used AND whether they are one- or two-sided<br>*Only common tests should be described solely by name; describe more complex techniques in the Methods section.*
☒	☐	A description of all covariates tested
☒	☐	A description of any assumptions or corrections, such as tests of normality and adjustment for multiple comparisons
☐	☒	A full description of the statistical parameters including central tendency (e.g. means) or other basic estimates (e.g. regression coefficient) AND variation (e.g. standard deviation) or associated estimates of uncertainty (e.g. confidence intervals)
☐	☒	For null hypothesis testing, the test statistic (e.g. *F*, *t*, *r*) with confidence intervals, effect sizes, degrees of freedom and *P* value noted<br>*Give P values as exact values whenever suitable.*
☒	☐	For Bayesian analysis, information on the choice of priors and Markov chain Monte Carlo settings
☒	☐	For hierarchical and complex designs, identification of the appropriate level for tests and full reporting of outcomes
☐	☒	Estimates of effect sizes (e.g. Cohen's *d*, Pearson's *r*), indicating how they were calculated

Our web collection on statistics for biologists contains articles on many of the points above.

Software and code

Policy information about availability of computer code

Data collection	ChemDraw 22 (chemical structures)<br>FACSDiva (modified CAPA assay)<br>Zeiss Zen v3.8-1 (Fluorescence microscopy)<br>Leica Application Suite X (confocal microscopy)<br>BioRad Image Lab 6 (Western Blots)<br>LabChart 8 (transepithelial short-circuit current measurements)<br>MassLynx v4.2, Acquity Console (HR-MS)
Data analysis	GraphPad Prism 10 (graphs, statistical analysis)<br>FIJI (Image analysis)<br>BioRad Image Lab 6 (Western Blots)<br>FlowJo 10.6.1-1 (flow cytometry)<br>MassLynx v4.2 (HR-MS)

For manuscripts utilizing custom algorithms or software that are central to the research but not yet described in published literature, software must be made available to editors and reviewers. We strongly encourage code deposition in a community repository (e.g. GitHub). See the Nature Portfolio guidelines for submitting code & software for further information.

Data

Policy information about availability of data

All manuscripts must include a data availability statement. This statement should provide the following information, where applicable:

- Accession codes, unique identifiers, or web links for publicly available datasets
- A description of any restrictions on data availability
- For clinical datasets or third party data, please ensure that the statement adheres to our policy

All experimental data, materials and methods, analytical procedures, cell assays, copies of spectra are available in the Main text and in the electronic supplementary information (ESI). Raw imaging data is available upon request

Research involving human participants, their data, or biological material

Policy information about studies with human participants or human data. See also policy information about sex, gender (identity/presentation), and sexual orientation and race, ethnicity and racism.

Reporting on sex and gender	Nasal epithelial cultures from healthy individuals were obtained from three donors, comprising two males and one female. Nasal epithelial cultures derived from cystic fibrosis (CF) patients homozygous for the F508del mutation included two female donors and one male donor.
Reporting on race, ethnicity, or other socially relevant groupings	We do not collect data on race, ethnicity, or other groupings.
Population characteristics	Healthy individuals aged 28, 30, and 34 years were non-smokers asymptomatic for respiratory disease at the time of nasal brushing collection.<br>Patients with cystic fibrosis homozygous for the F508del allele, aged 20, 20, and 28 years, exhibited clinical manifestations consistent with CF-related lung disease.
Recruitment	Written informed consent was obtained from all participants.
Ethics oversight	The study was approved by the ethics committee of the Charité - Universitätsmedizin Berlin (EA2/161/20). Written informed consent was obtained from all participants.

Note that full information on the approval of the study protocol must also be provided in the manuscript.

Field-specific reporting

Please select the one below that is the best fit for your research. If you are not sure, read the appropriate sections before making your selection.

☒ Life sciences ☐ Behavioural & social sciences ☐ Ecological, evolutionary & environmental sciences

For a reference copy of the document with all sections, see nature.com/documents/nr-reporting-summary-flat.pdf

Life sciences study design

All studies must disclose on these points even when the disclosure is negative.

Sample size	Sample sizes were determined based on practical considerations such as anticipated effect sizes and data variability.
Data exclusions	Full, uncropped Western blot images are presented, with wells excluded due to insufficient quality or signal artifacts clearly indicated.
Replication	With the exception of the determination of the cellular concentration, all experiments were conducted with biological replicates to ensure reproducibility. Imaging experiments were repeated at least once with similar results.
Randomization	All samples were randomly assigned to treatment groups.
Blinding	not applicable

Reporting for specific materials, systems and methods

We require information from authors about some types of materials, experimental systems and methods used in many studies. Here, indicate whether each material, system or method listed is relevant to your study. If you are not sure if a list item applies to your research, read the appropriate section before selecting a response.

Materials & experimental systems

n/a	Involved in the study
☐	☒ Antibodies
☐	☒ Eukaryotic cell lines
☒	☐ Palaeontology and archaeology
☒	☐ Animals and other organisms
☒	☐ Clinical data
☒	☐ Dual use research of concern
☒	☐ Plants

Methods

n/a	Involved in the study
☒	☐ ChIP-seq
☐	☒ Flow cytometry
☒	☐ MRI-based neuroimaging

Antibodies

Antibodies used	CFTR Monoclonal Antibody (CF3) (Invitrogen, #MA1-935, LOT: XL366836), Mouse IgM Isotype Control (11E10) (eBioscience™, Invitrogen, #14-4752-82,), Goat anti-Mouse IgM (Heavy chain) Alexa Fluor™ 647 (Invitrogen, #A21238, LOT: 2566350), CFTR antibody 596 (CFTR Antibodies Distribution Program of the Cystic Fibrosis Foundation, LOT: 596TJ10028520240213), horseradish peroxidase (HRP)-conjugated goat polyclonal anti-mouse antibody (Dako Denmark, P0047, LOT: 41236467), β-actin antibody (Sigma, A1978 LOT: 0000086303).
Validation	antibody profiles are from online databases

Eukaryotic cell lines

Policy information about cell lines and Sex and Gender in Research

Cell line source(s)	HeLa: American Type Culture Collection (ATCC); HEK293T (HEK), A549: German Collection of Microorganisms and Cell Cultures; CFBE mCherry-Flag-F508del-CFTR: Amaral Group (BioISI – Biosystems & Integrative Sciences Institute); CFBE41o-: Dr. Eric J. Sorscher (University of Alabama, USA)
Authentication	Cell profiles are validated by manufacturers
Mycoplasma contamination	All cell lines were tested regularly for mycoplasma contamination. All tested cell lines were negative for mycoplama contamination
Commonly misidentified lines (See ICLAC register)	not applicable

Plants

Seed stocks	not applicable
Novel plant genotypes	not applicable
Authentication	not applicable

Flow Cytometry

Plots

Confirm that:

☒ The axis labels state the marker and fluorochrome used (e.g. CD4-FITC).

☒ The axis scales are clearly visible. Include numbers along axes only for bottom left plot of group (a 'group' is an analysis of identical markers).

☒ All plots are contour plots with outliers or pseudocolor plots.

☒ A numerical value for number of cells or percentage (with statistics) is provided.

Methodology

Sample preparation	Cells were harvested with accutased, collected and washed with PBS 2x. After centrifugation, cells were stained with primary antibody and secondary antibody and resuspended in PBS before FACS analysis
Instrument	BD LSRFortessa
Software	FACSDIva, FlowJo 10.6.1-1
Cell population abundance	10,000 cells were collected for analysis.
Gating strategy	SSC vs. FSC (area) gating of live population. Mean fluorescence intensity of ACS (CFTR) intensity histogram was determined. For nanobody treated samples additional gating to exclude population without uptake was performed: a medium control sample was used to determine FITC negative population. Only FITC positive population was used.

☒ Tick this box to confirm that a figure exemplifying the gating strategy is provided in the Supplementary Information.
