# Supplementary figures and images for: A cell-permeable nanobody to restore F508del cystic fibrosis transmembrane conductance regulator activity
Source: Nat Chem Biol. 2026 Apr 17;22(7):1155–64. doi: 10.1038/s41589-026-02199-w (PMC13303082; doi:10.1038/s41589-026-02199-w)

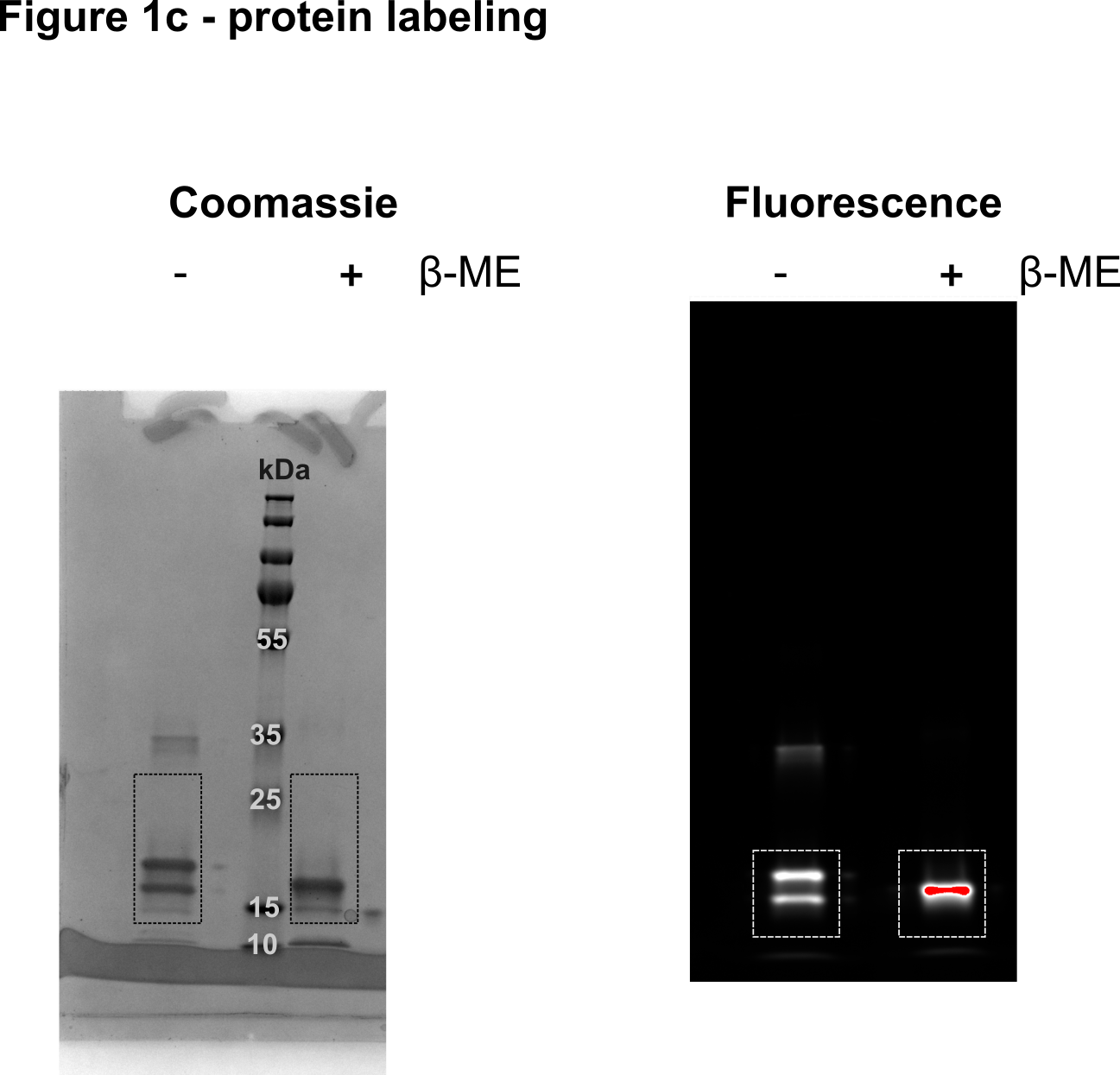

Supplement: Supplementary file 3 — Unprocessed gel. [file 41589_2026_2199_MOESM3_ESM.tiff]

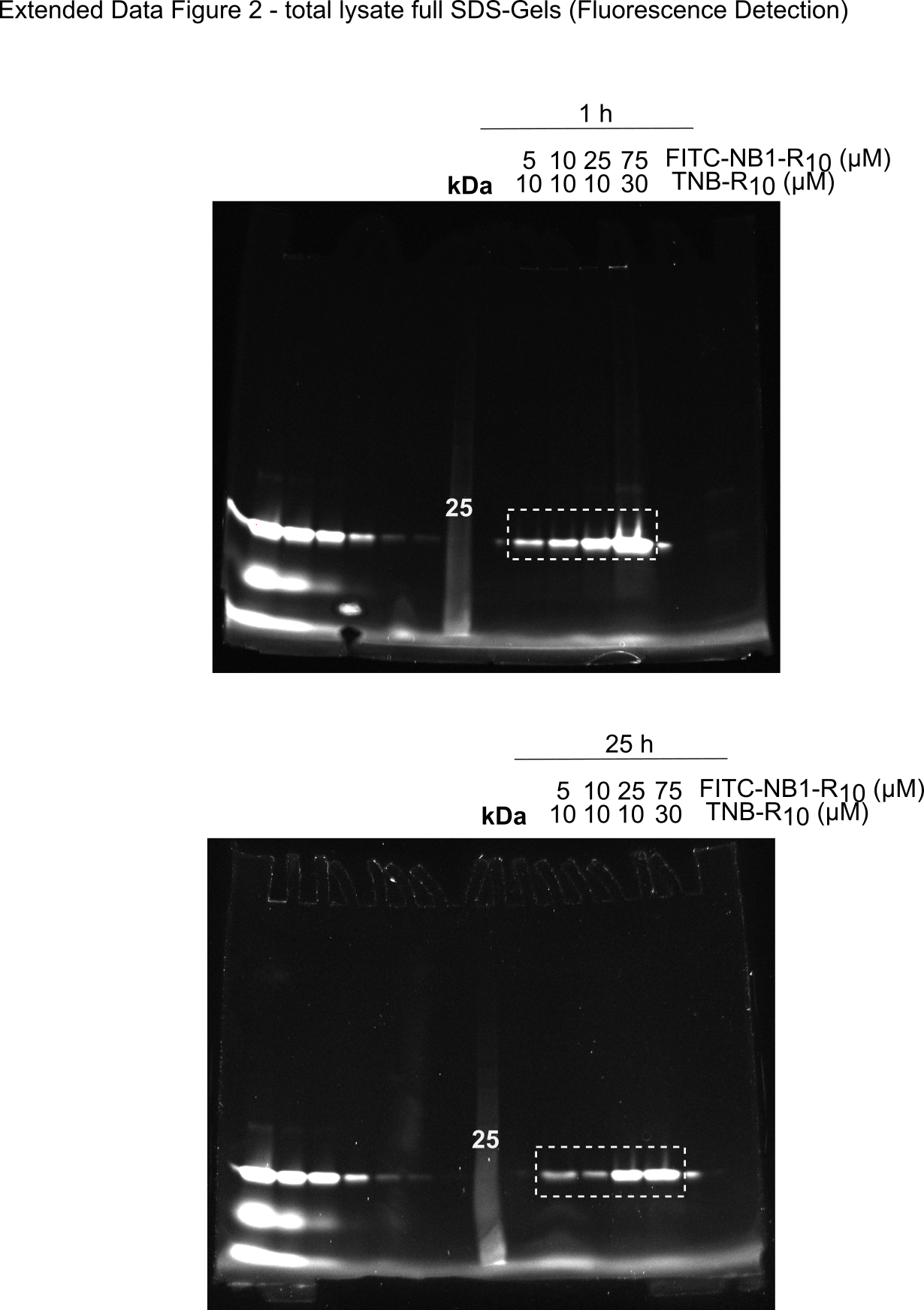

Supplement: Supplementary file 7 — Unprocessed gels. [file 41589_2026_2199_MOESM7_ESM.tiff]

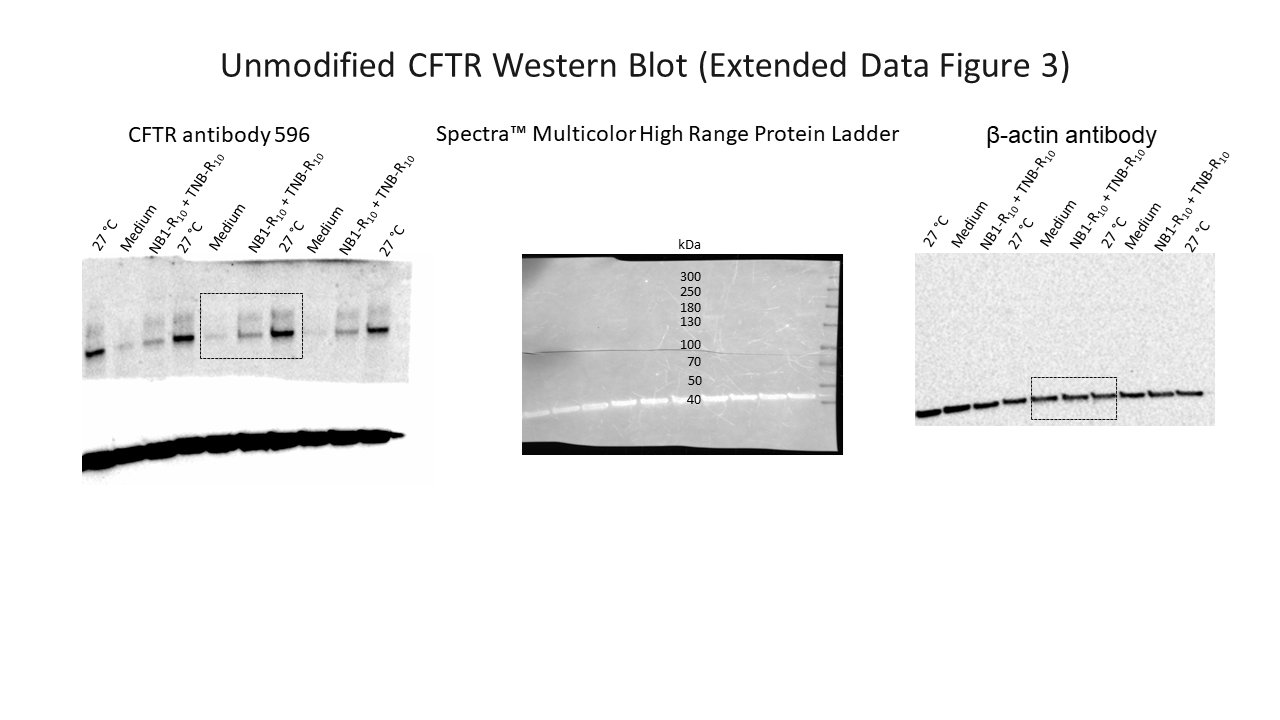

Supplement: Supplementary file 8 — Unprocessed western blots. [file 41589_2026_2199_MOESM8_ESM.tif]
